# Solid Phase Microextraction and Related Techniques for Drugs in Biological Samples

**DOI:** 10.1155/2014/921350

**Published:** 2014-02-13

**Authors:** Mohammad Mahdi Moein, Rana Said, Fatma Bassyouni, Mohamed Abdel-Rehim

**Affiliations:** ^1^Department of Chemistry, Amirkabir University of Technology, Tehran, Iran; ^2^Department of Analytical Chemistry, Stockholm University, SE10691 Stockholm, Sweden; ^3^National Research Center of Egypt, Cairo 12622, Egypt

## Abstract

In drug discovery and development, the quantification of drugs in biological samples is an important task for the determination of the physiological performance of the investigated drugs. After sampling, the next step in the analytical process is sample preparation. Because of the low concentration levels of drug in plasma and the variety of the metabolites, the selected extraction technique should be virtually exhaustive. Recent developments of sample handling techniques are directed, from one side, toward automatization and online coupling of sample preparation units. The primary objective of this review is to present the recent developments in microextraction sample preparation methods for analysis of drugs in biological fluids. Microextraction techniques allow for less consumption of solvent, reagents, and packing materials, and small sample volumes can be used. In this review the use of solid phase microextraction (SPME), microextraction in packed sorbent (MEPS), and stir-bar sorbtive extraction (SBSE) in drug analysis will be discussed. In addition, the use of new sorbents such as monoliths and molecularly imprinted polymers will be presented.

## 1. Introduction

Nowadays, the analytical instrumentations can provide a high resolution separation and low detection limits, down to picograms or below; the whole progressive analytical process can be wasted if an unsuitable sample preparation method has been employed. Thus, sample preparation is of crucial importance for the analysis of drugs in biological samples. The role of sample preparation is to remove interferences and analyte preconcentration, converting the analytes to suitable form for separation and detection. If an unsuitable sample preparation method has been employed before the injection, the whole analytical process may be wasted. Because of the low concentration levels of drug in plasma and the variety of the metabolites, the selected extraction technique should be virtually exhaustive. In recent years, solid phase microextraction (SPME) has been used by many researchers ever since it emerged in the early 1990s [1]. SPME technique has been widely used in many different areas such as environmental analysis [2], food analysis [3], bioanalysis [4], drug monitoring [5], and toxicology [6]. SPME can be used as direct immersion of the fiber into the sample to extract the analytes, or introduction of the fiber in the sample headspace to extract volatile compounds that are partitioned between gaseous and liquid phases. Many factors, such as pH, temperature, salt concentration, and stirring, affect the equilibrium constant and equilibration time [7–16].

Another microextraction related technique is stir-bar sorptive extraction (SBSE) which is an extraction technique for enrichment of volatile and semivolatile organic compounds having high extraction efficiency compared to SPME but has longer extraction time. The technique has been applied effectively in environmental analysis mainly with gas chromatography-mass spectrometry (GC-MS). In addition, the SBSE technique was applied to some drugs in biological samples in combination with GC-MS [17].

Recent development in solid phase microextraction related techniques is microextraction by packed sorbent (MEPS). MEPS is the miniaturization of conventional SPE and can be connected online to GC or LC without any modifications [18–22]. The extraction steps in MEPS are the same as in standard SPE—extraction, loading, washing, and elution—and these have to be optimized to obtain the highest analyte recovery. In MEPS, solvent and sample volumes are significantly reduced compared to SPE. The MEPS technique has been used to extract a wide range of drugs and metabolites in biological fluids such as urine, plasma, and blood [22].

This review presents recent developments of sample preparation in drug bioanalysis of complex fluids using SPME, SBSE, and MEPS.

## 2. Solid Phase Microextraction (SPME) for Drug Analysis

In SPME, the extraction is based on the partitioning of the analyte between the organic phase on the fused silica fibre and the matrix. Many factors, such as pH, temperature, salt concentration, and stirring, affect the equilibrium constant and the equilibration time [1]. Fibre lifetime is a significant issue. SPME fibre is quite sensitive to complex matrix such as plasma. In addition, type of polymer, temperature, duration, and additives coming from the sample solution influence the stability of the coating. It should be noted that additives such as sodium hydroxide and salt could catalyse polymer thermal degradation. In some bioanalytical studies, fibre life-time was decreased to about 20 samplings instead of 80 [8, 9]. In the two past decades, SPME as a sample preparation method for drug analysis has been used with various analysis methods. SPME was used with different separation techniques such as capillary gas chromatography (CGC) [23–30], GC/GC-MS [31–37], LC/LC-MS [38–50], GC-ICP-MS [51–57], and HPLC-UV/HPLC-MS [58–68].

### 2.1. Fiber and Monolithic In-Tube SPME

In tube solid phase microextraction (in-tube SPME) is a new format of SPME that can be coupled online to LC for automated analysis of less volatile and polar compounds like drug metabolites. This technique was used for the determination of drugs and metabolites in different biological matrices like urine, plasma and cell culture media from in vitro assays [69–71]. In addition fiber in-tube SPME online with capillary electrophoresis (CE) was used for the analysis of amitriptyline, imipramine, nortriptyline, and desipramine in human urine samples [72]. In this work, two types of Zylon fiber were used. One is high modulus (HM) with heat treatment and the other is regular (AS) without heat treatment after spinning the fibers. In addition, DB-5 capillary of 10 mm length was packed with a fiber with the same length. In order to investigate the effect of the fiber and the capillary coating on extraction efficiency, various kinds of extraction media were prepared as follows: HM fiber packed in a DB-5 capillary, HM/DB-5; AS fiber packed in a DB-5 capillary, AS/DB-5; HM fiber packed in an uncoated fused-silica capillary, HM/FS; and only DB-5 capillary, DB-5. For the evaluation of the effect of the packing density on the extraction efficiency, 26% and 52% packed capillaries were prepared. The packing density has been calculated by using the average diameter of the fibers as 11.5 *μ*m. The former was packed with 123 filaments and the latter with 246 filaments in a 10 mm DB-5 capillary [72]. This method was then applied to the analysis of amitriptyline in human urine and the results showed that the hyphenated system would be a powerful tool for the analysis of analytes in biological matrices, DB-5 capillary was cut to 10 mm length and the fiber of the same length was packed into this capillary. In order to investigate the effect of the fiber and the capillary coating on extraction efficiency, various kinds of extraction media were prepared as follows: HM fiber packed in a DB-5 capillary, HM/DB-5; AS fiber packed in a DB-5 capillary, AS/DB-5; HM fiber packed in an uncoated fused-silica capillary, HM/FS; and only DB-5 capillary, DB-5. For the evaluation of the effect of the packing density on the extraction efficiency, 26 and 52% packed capillaries were prepared. The packing density has been calculated by using the average diameter of the fibers as 11.5 *μ*m. The former was packed with 123 filaments and the latter with 246 filaments in a 10 mm DB-5 capillary. The density is based on the volume ratio between the space and the filled part of the inner capillary in which the fibers were packed. The running buffer was composed of 20 mM Na2HPO4 buffer (pH 9.3), 0.6 mM-cyclodextrin, and 20% acetonitrile.

**Figure 1:**
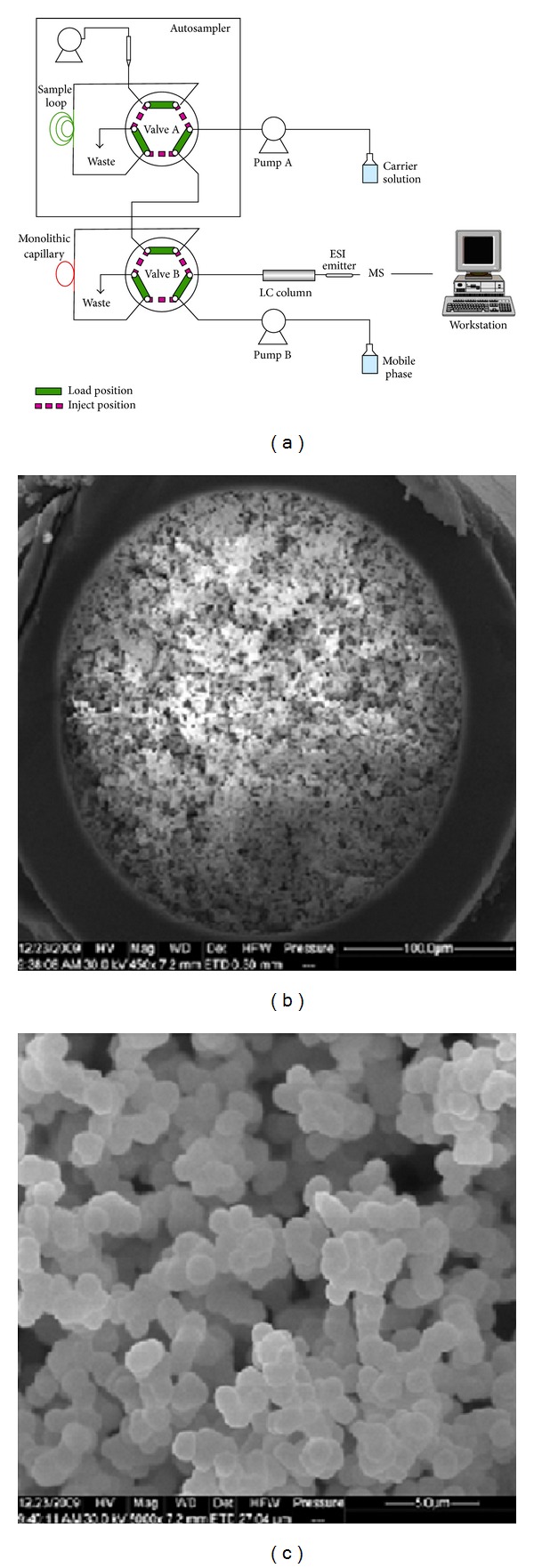
Construction of automated in-tube SPME-HPLC/MS system and Scanning electron microscope images of the cross section of the hybrid silica monolith: wide view (b) and close-up view (c) [74].

Monolith in-tube SPME is another approach for drug analysis in complex matrix. Different strategies were developed for preparation of monolithic in tube SPME (Figure 1). A hybrid organic-inorganic silica monolith with ceyanoethyl functional groups was synthesized by hydrolysis and polycondensation of precursors via a two-step catalytic sol-gel process that was used as a sorbent for in-tube SPME [73, 74]. Briefly, fused-silica capillaries (I.D. 250 *μ*m) were activated with 1 M NaOH and then 1 M HCl. After rinsing with double distilled water, they were dried at 160°C under N_2_ flow for 5 h. The hybrid monolith was synthesized by hydrolysis and polycondensation of precursors via a two-step catalytic sol-gel process. The optimal preparation conditions were as follows: 180 *μ*L of methanol, 25 *μ*L of 2 M acetic acid, 110 *μ*L off CN-TEOS, and 110 *μ*L of TEOS were mixed in a 1.5 mL Eppendorf vial. After thorough vortexing, the mixture was left for hydrolysis at 60°C for 5 h. After cooling to room temperature, 10 mg of N-dodecylamine was added to the solution. Then the pretreated capillary was filled to a certain length with the sol by a syringe. The capillary was sealed at both ends with silicone rubber and then was allowed to further react at 40°C for 15 h. Subsequently, the capillary was rinsed with ethanol to remove the N-dodecylamine and soluble hydrolysis products and then dried at 60°C for 48 h. The total and effective lengths of the hybrid silica monolith were 20 and 15 cm, respectively [74].

As it is shown in Figure 1, four capillaries are connected in the modified cross connector to build the online fiber-in-tube SPME-CE system. To minimize the band broadening effects, the gap between the separation capillaries must be strictly decreased. Therefore, the capillaries between the two buffer reservoirs were connected using a microscope until the gap between the capillaries was less than 10 *μ*m. In addition, the SEM picture (Figure 1) showed that the monolith is attached tightly to the inner-wall of the capillary. The flow-through pores size distribution determined by mercury porosimeter was around 4 m with a narrow size distribution, which results in high permeability and favourable mass transfer in extraction applications.

**Figure 2:**
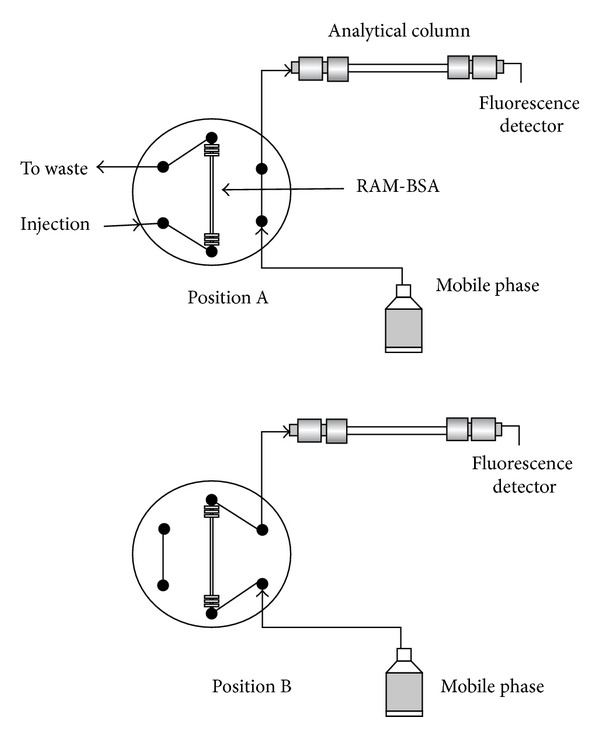
Scheme of the operation mode of the six-port switching valve in the RAM in-tube SPME developed method [76].

In another work, a restricted access material (RAM) was employed for preparation of a lab-made biocompatible in-tube SPME capillary that enables the direct injection of biological fluids as well as the simultaneous exclusion of macromolecules by chemical diffusion barrier and drug preconcentration [75, 76]. In this work, silica particles (C18–45 *μ*m) were slurried in methanol and packed into 50 mm (length) of polyether ether ketone (PEEK) tubing (I.D. 0.02 inch) and then the capillary column was capped at both ends by a 1/16 in. (1 in. = 2.54 cm) zero-volume union fitted with a 10 *μ*m frit. After this procedure, the capillary was conditioned with phosphate buffer (0.05 mol L^−1^, pH 6.0) at a flow-rate of 1.0 mL min^−1^ for 20 min. Initially, 50 mL phosphate buffer solution (0.05 mol L^−1^, pH 6.0) was percolated through the capillary at a flow rate of 1.0 mL min^−1^, followed by 25 mL Bovine serum albumin solution 1.0 mg mL^−1^ (prepared in phosphate buffer solution) and by 25 mL glutaraldehyde solution (25%, v/v). After 5 h, the columns were washed with 10 mL sodium borohydride solution (1.0 mg mL^−1^) and then with 60 mL water. The RAM-BSA column was stored in phosphate buffer solution (0.05 mol L^−1^, pH 7.4) at 4°C. The schematic structure of RAM is shown in Figure 2.

**Figure 3:**
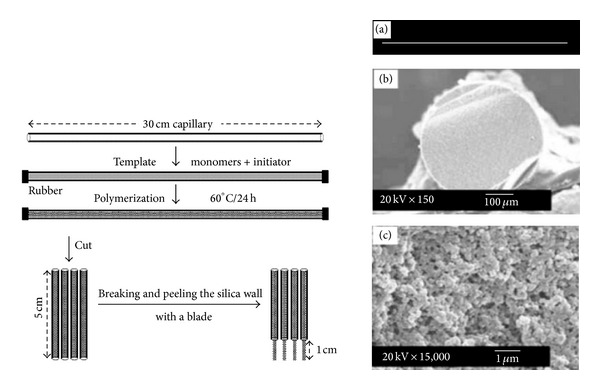
Schematic illustration of MIP preparation procedure [78].

In addition, monolithic molecular imprinted polymer (MIP) fiber based solid phase microextraction (SPME) was developed for selective and sensitive determination of different drugs and biomarkers in biological samples [77, 78]. In situ polymerization of silica capillary mold using E as template was reported and in some studies the MIP fibers are preparedand each fiber could be used for about 50 extraction-cycles without any significant decrease in extraction capacity.  Figure 3 illustrated the MIP strategy for preparation of MIP in tube SPME fiber.

**Figure 4:**
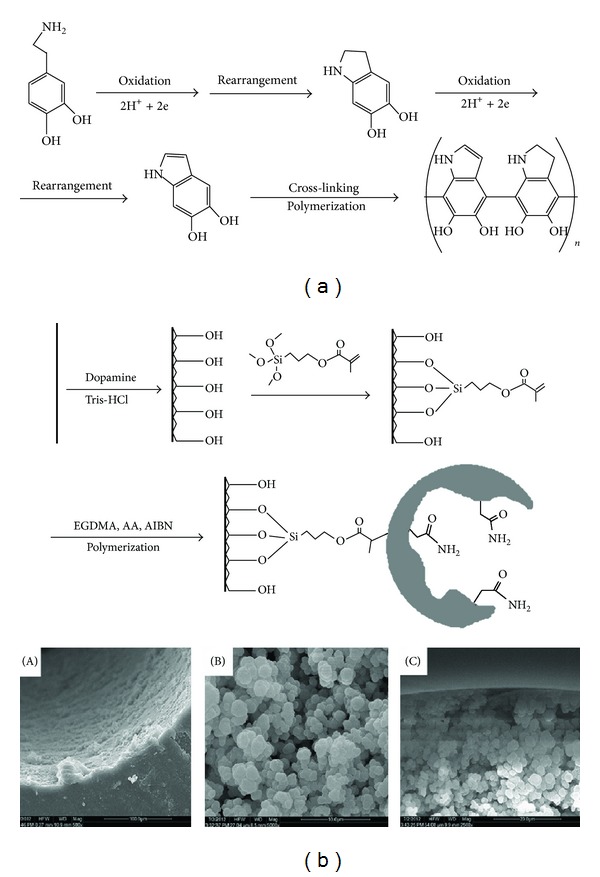
Schematic of (a) the formation of polydopamine layer and (b) modification of PEEK tube and preparation of poly(AA-EGDMA) monolith and Scanning electron micrograph of (A) polydopamine layer on the inner wall of PEEK tube, (B) poly(AA-EGDMA) monolith, and (C) interface of inner wall of PEEK tube and polymer monolith [79].

Other types of monolithic in tube SPME were prepared by different kinds of monomer and cross-linker mixtures, such as poly(acrylamide-ethylene glycol dimethacrylate). Poly(AA-EGDMA) monolith was selected as sorbent for SPME of three protoberberine alkaloids (Figure 4). Briefly, AA was weighed and put in a 1 mL screw capped glass vial, followed by adding isooctane, toluene, and methanol as porogen. After AA was completely dissolved, the cross-linker EGDMA and the initiator AIBN were added to the above solution. Ultrasonication was applied for 20 min to remove dissolved oxygen. Finally, the prepolymerization solution was introduced into the modified PEEK tube carefully, and then the PEEK tube was sealed and put into a water bath for polymerization (60°C, 3 h). After polymerization, the monolith was washed with acetonitrile to remove porogen and unreacted reagents [79], poly(meta acrylic acid-ethylene glycol) [80–82], and poly(4-vinylpyridine-co-ethylene dimethacrylate) [83]. Also, in-tube SPME/LC method was developed and validated for rifampicin interferon *α*_2a_ determination in plasma samples for therapeutic drug monitoring and in plasma samples and lidocaine and its Metabolite MEGX in plasma samples [84–86].

In summary, monolithic in-tube SPME was shown to be an appropriate method for drug metabolism studies and routine analysis or pharmacokinetics as the parent compound and main metabolites could be monitored in various matrices of interest.

### 2.2. Headspace Solid-Phase Microextraction (HS-SPME) in Drug Bioanalysis

HS-SPME, an alternative sample extraction technique, allows concentrating volatile and semivolatile analytes from the headspace above the sample on a coated fiber and to transfer the analytes from the fiber directly into the injector port of a GC without further manipulations [87].  Table 1 shows the different drugs and pharmaceutical components determined by HS-PME method.

**Figure 5:**
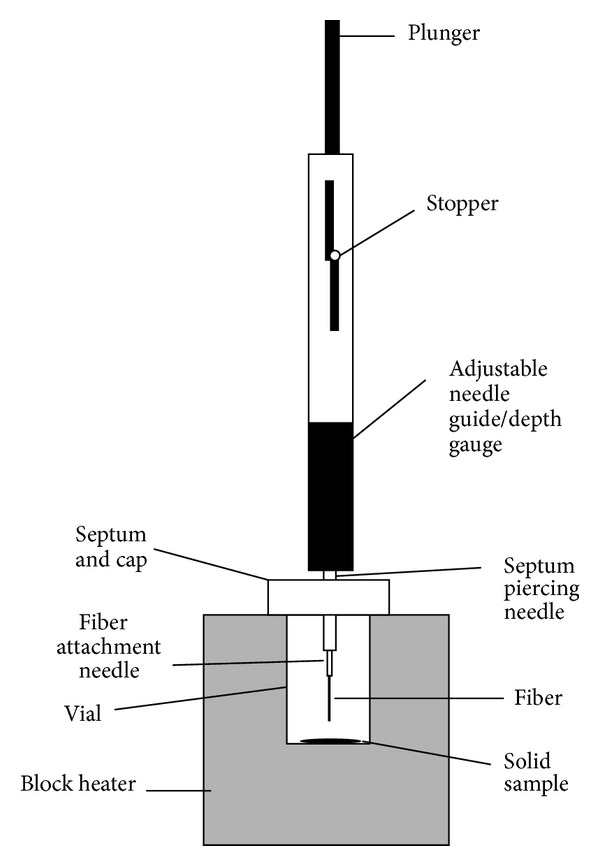
SPME high-temperature headspace sampling [88].

A schematic structure of HS-PME is shown in Figure 5 [88]. In some works commercial fibers were used. In addition, the sol-gel method was used for fiber preparation in HS-PME technique. In the following part, we will describe different kinds of precursors that were used in HSPME method.

### 2.3. Sol-Gel HS-SPME

The sol-gel process provides a useful method of preparing organic-inorganic hybrid materials through the hydrolysis and condensation of suitable metal alkoxides, particularly, silicone alkoxide, which readily allows forming three-dimensional (3D) network under relatively mild conditions [89].

**Figure 6:**
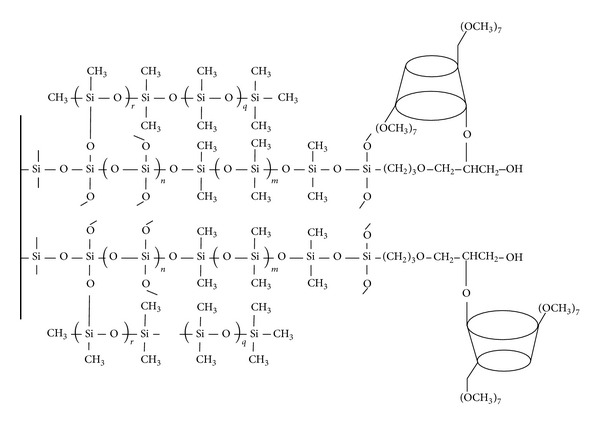
The chemical structure of the DM-*β*-CD/OH-TSO coating [34].

Table 2 lists the names and chemical structures of the principal ingredients of the coating solution used. The sol-gel process may involve mainly several parts as follows: (1) ring-opening polymerization between KH-560 and DM-*β*-CD; (2) hydrolysis and polycondensation among the product of (1), TEOS, and OH-TSO to generate a 3D network; (3) chemical anchoring of the polymeric networks to the outer surface of the fused-silica fiber; (4) deactivating residual silanol groups on the stationary phase with PMHS, aimed to reduce harmful adsorptive effects. Thus, a surface-bonded polymeric coating DM-*β*-CD/OH-TSO is formed as schematically represented in Figure 6 [90].

### 2.4. Molecularly Imprinted Polymers Solid Phase Microextraction (MIPs-SPME)

Molecularly imprinted polymers (MIPs) have proven to be useful materials in analytical chemistry. MIPs are cross-linked synthetic polymers obtained by copolymerizing a monomer with a cross-linker in the presence of a template molecule. After polymerization, the template is removed from the porous network by washing, leaving cavities in the polymeric matrix that are complementary in size, shape, and chemical functionality to the template. Thus, the imprinted polymer is able to rebind selectively the analyte (the template) under certain experimental conditions [91].

**Figure 7:**
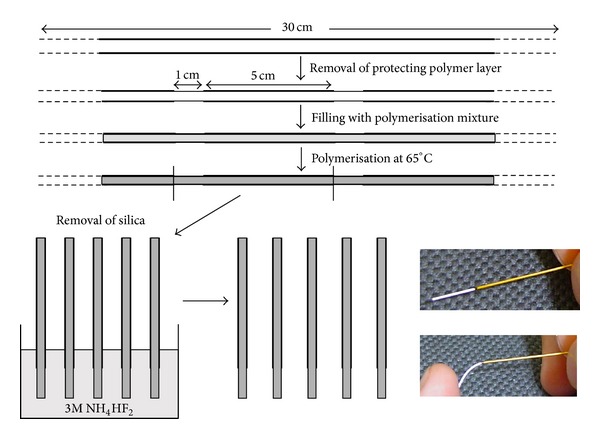
Preparation of molecularly imprinted fiber [91].

**Figure 8:**
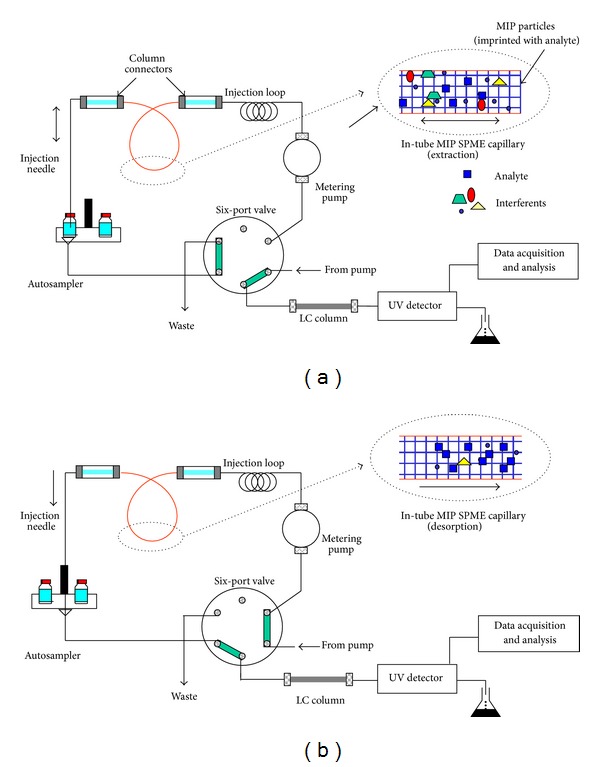
Schematic representation of in-tube MIP SPME configuration. (a) Load position (extraction); (b) injection position (desorption) [92].

Accordingly, the combination of molecular imprinting and SPME would ideally provide a powerful analytical tool with the characteristics of both technologies, simplicity, flexibility, and selectivity. There are two strategies in this field; the easiest way for combining both technologies was proposed by mullet [92], which consisted of packing a capillary with the MIP particles for in-tube SPME and was used for the selective determination of propranolol in serum samples. The developed method was successfully applied and the advantages of in-tube SPME were obvious (high enrichment factors provided by multiple draw/eject cycles, ease of automation, and fast operation). However, this methodology is not free of some important drawbacks such as the lack of compatibility between the solvent needed to desorb analytes from the MIP and the mobile phase used (typical drawback of online MISPE protocols) and the necessity of extra instrumentation (pump, multiport valves). Thus, the preparation of silica fibers coated with a MIP to perform SPME would be the best option and different works have been developed in this field [91]. Figures 7 and 8 show schematic setup of these two strategies.

Most papers that have been developed in MIP-SPME field are about fiber preparation for separation of different valuable targets of complex media such as Clenbuterol and Structural Analogues [93], triazines [94], diacetylmorphine and analogous compounds [95], Prometryn [96], bisphenol A [97, 98], anabolic steroids [99], 2,2′-bipyridine [100], antibiotic drugs [101], and sulfamethazine [102].

#### 2.4.1. Preparation of MIP-Coated Fibers (MIP-CF)

**Figure 9:**
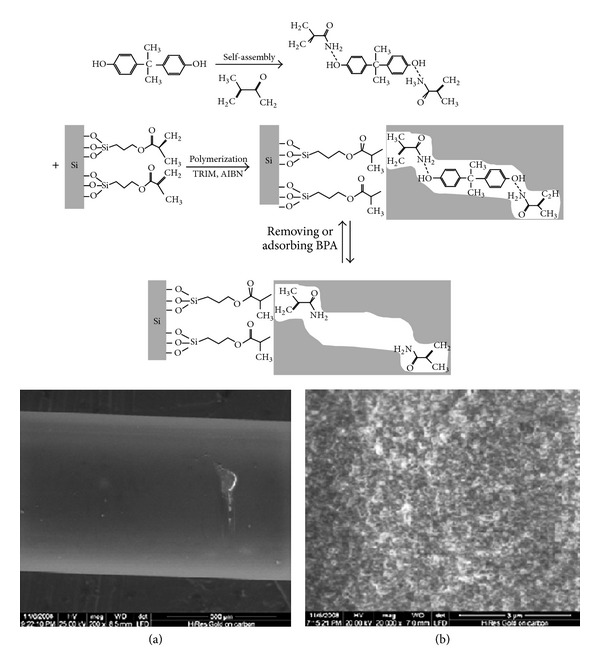
The preparation process of BPA-imprinted MIP coating on the silylated wall of the silica capillaries. Schematic representation of BPA-imprinted MIP coating on the silylated wall of the silica capillaries and Scanning electron micrographs of MIP coating of the fiber. (a) ×200, (b) ×20000 [97].

SPME conditions based on the MIP-coated fibers are valuable methods that developed in recent years. As simple approach for preparation of bisphenol A (BPA) MIP-coated SPME fibers a capillary was inserted into a larger bore capillary to form a sleeve as mold [97]. The prepolymer solution, which comprised BPA, acrylamide (AM), 3-(trimethoxysilyl) propyl methacrylate (TRIM), AIBN, and ACN, was introduced into the interspace between the two capillaries, followed by polymerization under UV photoirradiation (Figure 9). The larger bore capillary was etched away with hydrofluoric acid after the polymerization. This approach showed that this very simple method could become a routine preparation procedure for MIP-coated fibers. The MIP coating on the silica fibers was homogeneous and porous and showed good mechanical and chemical stability. According to the result as of this work, it was demonstrated that the MIP-coated fibers had better adsorption/desorption kinetics compared with the monolith MIP fiber. Under the optimized SPME conditions, selective extraction of BPA from standard mixture aqueous sample was feasible with the MIP-coated fibers.

## 3. Stir-Bar Sorptive Extraction (SBSE)

Since SBSE was developed in 1999 [17], it has already shown significance among the sorptive extraction techniques. SBSE and SPME are microextraction techniques with low, or even no, consumption of organic solvents. The analytes are extracted from the matrix into the polymer coating immobilized on a glass tube with a magnetic core. Rapid molecular-recognition equilibrium between adsorption and desorption can be established, since sampling is performed simultaneously with the stirring. As a result, competitive sorption from an additional stirrer (e.g., magnet essential for the SPME technique) can also be avoided [103].

The main differences between the two techniques are the design of the extraction system and the amount of the sorbent material. The sorbent materials are similar, although till today the availability of commercial SBSE materials is rather limited [104]. Contrary to SPME, quantitative recoveries are often achievable with SBSE due to the clearly higher sample capacity. SBSE can also be employed for the extraction of relatively polar compounds. Quantitative extraction can be achieved for solutes with log *K*_o/w_ values of ca. 4, and reasonable efficiencies are obtained for solutes with log *K*_o/w_ values above 3 [105] For highly polar compounds, similar approaches as for SPME can be applied (i.e., derivatisation). Same as SPME in SBSE various parameters such as type and thickness of the coating, extraction time, sample properties (pH, ionic strength), agitation, temperature, and analyte desorption could be evaluated. The extraction time is typically longer than in SPME, because the amount of coating is greater and it takes longer to reach equilibrium. The analyte desorption is more critical for SBSE than for SPME, likewise due to the greater amount of coating. A high flow rate of gas (up to 100 mL min^−1^) is recommended for fast desorption of analytes during thermal desorption [105].

### 3.1. Sol-Gel Technology in Stir-Bar Sorptive Extraction

For the first time, Liu et al. [106] used sol-gel technology in stir-bars to produce a partially hydroxyterminated-PDMS coated stir-bar, which was used for extracting a group of PAHs and organophosphorous compounds.

**Figure 10:**
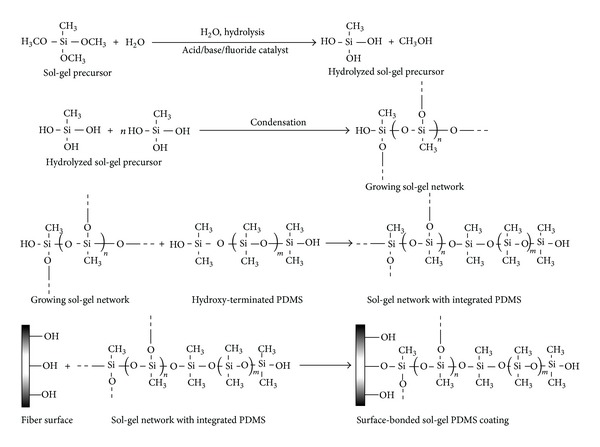
Chemical reactions involved in the synthesis of surface-bonded sol-gel hybrid organic-inorganic polymeric network [107].

The sol-gel process offers a convenient, versatile pathway for preparing advanced inorganic and organic-inorganic hybrid material systems, with tunable porosity, selectivity, and thermal and chemical stability. The schematic of sol-gel reactions is shown in Figure 10 (where methyltrimethoxysilane (MTMOS) and hydroxy-terminated polydimethylsiloxane (PDMS) are shown to represent sol-gel precursor and sol-gel active organic polymer, resp.) [107].

Despite its numerous advantages over conventional SPME fibers, SBSE also suffers from serious limitations [107] as follows:

1. limited number of commercially-available coatings;
2. coating is not chemically bonded to the substrate leading to the possibility of bleeding at even relatively low temperature during thermal desorption and transfer of the extracted analytes from the stir-bar to the GC system;
3. coating is vulnerable to washing away if proper solvent is not used during solvent desorption;
4. thermal desorption requires an expensive thermal desorption unit;
5. thick, highly viscous polymeric sorbents used on the stir-bar require hours to reach the extraction equilibrium;
6. there is a need for a relatively high volume of back extraction solvent, which evidently dilutes the preconcentrated analytes.

High porous sol-gel PDMS coated stir-bar with 30 *μ*m coating thickness was developed by Liu et al. [106]. The coating was found thermally stable up to 300°C. The sol-gel PDMS coated stir-bars were tested for the extraction of *n*-alkanes, PAHs, and organ phosphorus pesticides. The sol-gel PDMS coated stir-bar reached extraction equilibrium in less than 15 min. In addition, unlike commercial PDMS coated stir-bars, the sol-gel PDMS coated stir-bar is equally suitable for both polar and nonpolar analytes.

Different sorbents for SBSE sol-gel method were developed in recent years. A sol-gel PDMS/PVA coated stir-bar for the extraction of organophosphorus pesticides (OPPs) in honey samples was used [108]. The extracted analytes were back-extracted by solvent desorption. The back-extraction solvent, which contained the analyte(s) of interest, was then injected into the GC using large volume injection, followed by GC-FPD. The extraction performance of sol-gel CW/PDMS/PVA was compared with commercial PDMS stir-bar and Carboxen/PDMS SPME fiber using headspace extraction. Sol-gel CW/PDMS/PVA coated stir-bar demonstrated the highest sorption capacity and ~10 times higher sensitivity [109]. Also, a sol-gel PDMS/*β*-CD coating (30–150 *μ*m) for the extraction of polar compounds from different matrices (e.g., estrogens in environmental water and bisphenol A in drinking water) [110]. The same sol-gel sorbent (sol-gel PDMS/*β*-CD) was utilized for extracting brominated flame retardants from soil and dust samples employing ultrasound-assisted extraction followed by HPLC analysis [111]. SBSE performances were compared for four different sol-gel coatings including PDMS/b-CD, PDMS, CW/PDMS/PVA, and PDMS/PVA. The sol-gel PDMS/*β*-CD coated stir-bar was found to be the most efficient for the target compounds. In addition to superior extraction performance, sol-gel PDMS/*β*-CD coated stir-bars demonstrated excellent durability, and no discernible loss of extraction efficiency was observed even after 100 extraction cycles.

### 3.2. Molecularly-Imprinted Stir-Bar Sorptive Extraction (MI-SBSE)

**Figure 11:**
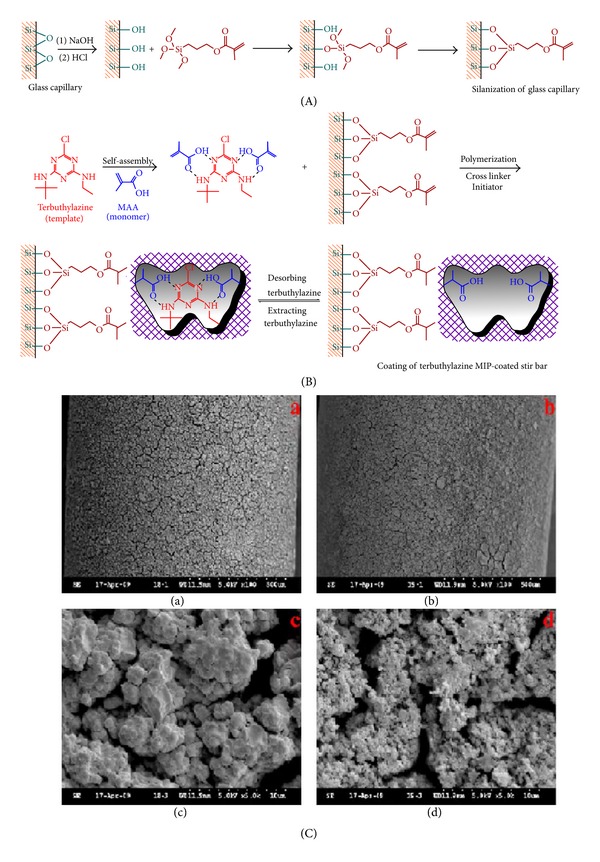
Schematic diagrams of the preparation of MIP-SBSE coating using terbuthylazine as template molecule and Scanning electron micrographs of the surface structure of the MIP- and NIP-coated stir-bar. (a) and (c) are the NIP coating for magnitude of 100 and 5000, respectively, (b) and (d) are the MIP coating for magnitude of 100 and 5000, respectively [114].

MI-SBSE is based on the partitioning of target analytes between a liquid sample and a stationary phase-coated stir-bar. Until now, only polydimethylsiloxane (PDMS) coated stir-bars are commercially available, restricting the range of applications to the extraction of hydrophobic compounds (organochlorine and organophosphorus pesticides) due to the polar character of PDMS. Besides, the MIP-coated stir-bars showed not only the expected high selectivity but also rapid equilibrium adsorption, thanks to the porous nature of the imprinted polymer obtained combined with a suitable thickness of coated polymer film (*μ*160–180 lm) [112]. More recently, the use of MIP-coated stir-bars prepared by chemical bonding of the MIP to the stir-bar through silylation of the substrate surface and then multiple copolymerization reaction was proposed for the determination of various components in different samples [113–117]. The schematic diagrams of the preparation of MIP-SBSE coating using terbuthylazine as template molecule is shown in Figure 11 [114].

### 3.3. Stir-Bars Sorptive Extraction Based on Monolithic Material (SBSEM) and Molecularly Imprinted Polymer Monolith Microextraction (MIPMME)

**Figure 12:**
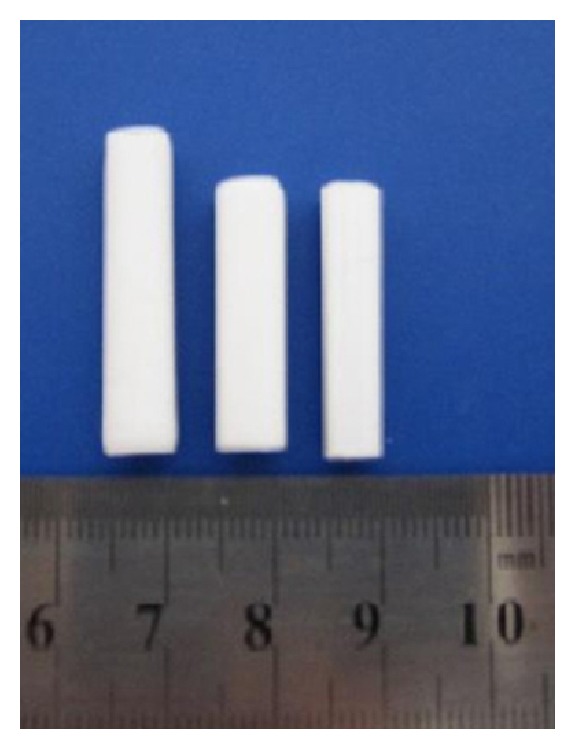
Photograph of different dimensions of SBSEM [118].

The preparation of monolithic materials is very simple just by polymerization of a monomer mixture with a porogen solvent, forming a porous polymer. In this way, Huang and Yuan developed monolithic material obtained by in situ copolymerization of octyl methacrylate and ethylene dimethacrylate in the presence of a porogen solvent containing 1-propanol, 1,4-butanediol, and water with azobisisobutyronitrile as the initiator [118]. The results demonstrate that prepared stir-bar was suitable for preconcentration of both apolar and polar analytes. The enrichment factors for phenanthrene, anthracene, and pyrene were 150, 134, and 189, respectively. The SBSEM shows good batch-to-batch reproducibility and good stability and can be reused at least 10 times for the extraction of polycyclic aromatic hydrocarbons in seawater (Figure 12).

In another in situ copolymerization approach, vinylpyrrolidone and divinylbenzene in the presence of a porogen solvent containing cyclohexanol and 1-dodecanol with azobisisobutyronitrile as initiator were used for SBSEM preparation [119]. Polycyclic aromatic hydrocarbons were used to investigate the extraction efficiencies of SBSEM for apolar analytes. Hormones, aromatic amines, and phenols were selected as test analytes to investigate the extraction efficiencies of SBSEM for weakly and strongly polar compounds. The results showed that the new SBSEM could enrich the above-mentioned organic compounds effectively. It is worthy to mention that the SBSEM can enrich some heavy metal ions, such as Cu^2+^, Pb^2+^, Cr^3+^, and Cd^2+^, through coordination adsorption.

In another case, poly(vinylpyridine-ethylene dimethacrylate) is used as SBSEM combined with high performance liquid chromatography with diode array detection under the optimized experimental conditions for analysis of target compounds in wastewater samples [120]. The method showed good linearity and repeatability, as well as advantages such as sensitivity, simplicity, low cost, and high feasibility.

## 4. Microextraction by Packed Sorbent (MEPS)

MEPS was recently introduced as a novel method for sample preparation, being a miniaturization of the conventional SPE technique, in which the sample volume, extraction, and washing solvents volumes are greatly reduced compared to SPE. MEPS differs from commercial SPE in that the packing is integrated directly into the syringe and not into a separate column. Moreover, the packed syringe can be used several times, more than 100 times using plasma or urine samples, whereas a conventional SPE column is used only once. MEPS can handle small sample volumes (10 *μ*L plasma, urine, or water) as well as large volumes (1000 *μ*L).

**Figure 13:**
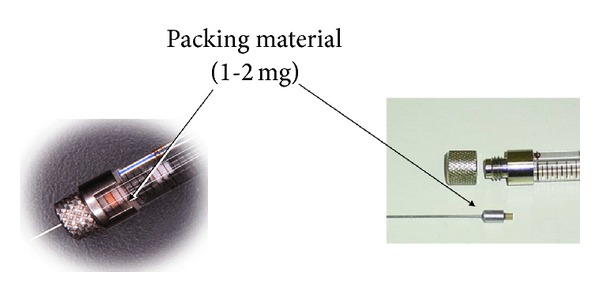
MEPS syringe with sorbent.

The extraction steps in MEPS are the same as in standard SPE extraction, washing, and elution and these have to be optimized to obtain the highest analyte recovery. In MEPS extraction procedures, additional steps for postcleaning and reconditioning have to be included to enable multiple uses of the MEPS sorbent. Blood samples require dilution of 20–25 times with water or acidic water, while 4-5 times dilution is needed for plasma samples. Using mixed-mode (anion-cation exchange), the sample pH has to be adjusted to produce charged analytes. MEPS was used with real samples from clinical institutions or pharmaceutical industry for established drugs and new drug candidature (Figure 13).

The superior performance of MEPS was recently illustrated by online LC-MS and GC-MS assays of drugs and metabolites in water, urine, plasma, and blood samples [18–22, 121–153]. The combination of MEPS and liquid chromatography mass spectrometry (LC-MS) is a good tool for the screening and determination of drugs and metabolites in blood, plasma, and urine samples. Mass spectrometry is presently one of the most powerful detection techniques, particularly in pharmaceutical analysis, where good selectivity and high sensitivity are often needed. MEPS significantly reduces the volume of solvent and sample needed. This approach to sample preparation is very promising for many reasons: (1) it is easy to use, (2) it is a fully automated online procedure, (3) it is rapid, and (4) the cost of analysis is minimal compared to conventional solid-phase extraction.

### 4.1. MEPS Extraction Procedures

Plasma samples were diluted four times while blood samples diluted 20 times with water.

*(i) Conditioning Step.* The sorbent was conditioned with 150 *μ*L methanol and subsequently with 150 *μ*L water.

*(ii) Sample Loading Step.* Sample can be loaded by multiple aspirates-dispenses cycles (5 × 100 *μ*L).

*(iii) Washing Step.* The sorbent was washed after sample loading by 150 *μ*L water.

*(iv) Elution of the Analytes.* Elution solution was organic solvent (≥60%).

The MEPS technique has been used to extract a wide range of analytes in different matrices (urine, plasma, and blood). Hence, several drugs such as local anesthetics and their metabolites, the anticancer drugs roscovitine, olomoucine, busulphan, cyclophosphamide, and AZD3409, the *β*-blockers acebutolol and metoprolol, the neurotransmitters dopamine and serotonin, methadone, and cocaine and cocaine metabolites have been extracted from biological samples such as blood, plasma, or urine samples using MEPS [18–22, 121–153].  Table 3 shows the application of MEPS technique in different matrices and various types of drug.

### 4.2. Molecularly Imprinted Polymer as Sorbent in MEPS

Molecularly imprinted polymer was used as extracting sorbent in MEPS for the simultaneous determination of four local anaesthetics (ropivacaine, lidovacaine, bupivacaine, and mepivacaine) in human plasma and urine samples have been evaluated. In comparison with protein precipitation, MIP-MEPS offer enrichment of analytes and elimination of interferences from matrix constituents. This may be important for increasing sensitivity and for robustness of the LC-MS system. Ion suppression was observed in protein precipitation method for lidocaine, ropivacaine, and mepivacaine. The matrix effect was more pronounced using protein precipitation. The matrix effect (ME) for these substances ranged from 50% to 146% using protein precipitation while it was less than 20% using MIP-MEPS. This suggests that cleaner extracted samples can be obtained with MEPS and that matrix related problems can be reduced [147]. In comparison with conventional SPE, the MEPS method can handle smaller sample volumes (10–100 *μ*L). This is mainly an advantage for samples from children.

### 4.3. Extraction of Proteins from Plasma by MEPS

MEPS technique online with LC-MS/MS was used for the quantification of SNSR receptors agonist peptide BAM8-22 and antagonist BAM22-8 in plasma samples [148]. MEPS-C8 was used and spiked plasma sample (125 *μ*L) was diluted (1 : 1) with 0.1% CHOOH in water; 50 *μ*L were drawn onto the MEPS-syringe three times. The sorbent was then washed once with 50 *μ*L of 5% methanol in water to remove other interferences. The analytes were then eluted by 40 *μ*L 0.25% ammonium hydroxide in methanol-water 95 : 5 (v/v) directly into LC injector. The calibration curve in plasma was in the range of 20.0–3045 nmol/L. The regression correlation coefficients for plasma samples were ≥0.99. The between-batch accuracy and precision for BAM8-22 ranged from −13 to −2.0% and 4.0 to 14%, respectively. Additionally, the accuracy and precision for BAM22-8 ranged from −13 to 7.0% and from 3.0 to 12%, respectively. The method was used for pharmacokinetic studies for BAMs in plasma samples [148]. MEPS technique provided significant advantages such as the speed and the simplicity of the sample-preparation process. Compared with other extraction techniques, such as protein precipitation and ultrafiltration, MEPS gave cleaner samples and higher recovery (>90%). The method had good accuracy and good precision within the studied calibration range. Furthermore, the method reduced the sample preparation time for BAMs (less than one minute per sample), which is of great importance to handle unstable analytes such as BAMs in plasma and blood samples. The method was applied to plasma samples from preclinical studies [148]. The potential savings in handling time reduced solvent use. The simplicity of MEPS technique will continue to attract interest among analytical chemists searching for improved analysis methods.

In summary, the significant advantages of MEPS are reduction in the amount of sorbent bed, solvents, and sample preparation times and reduction in carry-over effects.

## 5. Monolithic Packed 96-Tips for High Throughput Bioanalysis

Recently we introduced a 96-tips set packed with a plug of a monolithic adsorbent. Using such a set, it is possible to handle a 96-well plate in only 2 minutes [154]. Packed 96-tips sample preparation is a clean, highthroughput, and automated sample-preparation method. Samples are prepared in a 96-well plate format and the analytes adsorb onto the polymer-based monoliths in the extraction step. The next step purifies the sample by washing the sorbent with an appropriate washing solution. In a final step, the analytes were directly eluted into a 96-well plate using an appropriate solvent for the analytes and the subsequent instrumental analysis.

### 5.1. Preparation of Monolithic Plug in Tips

**Figure 14:**
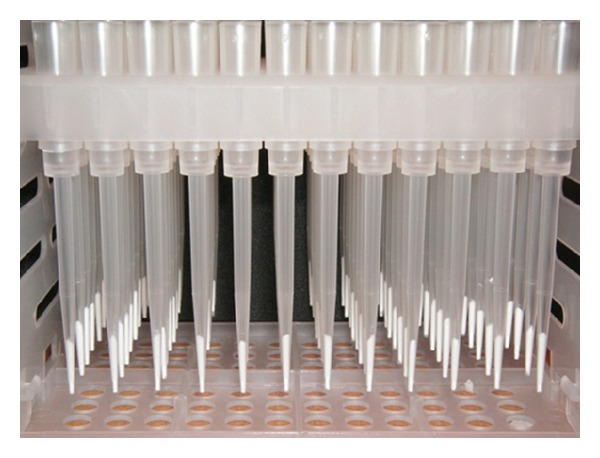
Monolithic packed 96-tips.

The polymerization mixture of methacrylate monoliths consists of a solution containing glycidyl methacrylate (20%), ethylene glycol dimethacrylate (15.5%), butyl methacrylate (3.5%), AIBN (1 wt% with respect to monomers), and 1-dodecanol (30%) and cyclohexanol (30%) was vortexed for 10 min and purged with nitrogen for 10 min in order to remove oxygen. The pipette tips were filled with about 8 mm (6-7 *μ*L) by the capillary action and placed vertically inside the polymerization apparatus. The polymerization using UV light at 254 nm was allowed to proceed first for 60 min with the sharp end of the tip down and at a distance to the lamp of 15 cm and then for 25 min with the sharp end up and at a distance of 5 cm to the lamp. After completion of polymerization, the tips were removed, inspected under microscope for bubbles, and washed with acetone to remove the porogenic solvents and other compounds remaining in the monolith (Figure 14).

The key aspect of the monolithic phase is that monolithic material can offer both good binding capacity and low back-pressure properties compared to, for example, silica phases. Using this device, a 96-well plate could be handled in 2–4 minutes.

### 5.2. Monolithic Packed 96-Tips Application in Bioanalysis

Evaluation of monolithic packed 96-tips for the extraction of drugs [154–159] such as anticancer drugs (busulphan, cyclophosphamide, roscovitine), *β*-blocker drugs (metoprolol, pindolol), and local anesthetics (lidocaine, ropivacaine, bupivacaine) from human plasma or blood samples has been developed and validated (Table 4). Utilizing plasma samples, the tips could be used several times (5 times) and still get good results. Utilizing blood samples, packed tips could only be used once. The results showed that the method is selective and accurate. It was shown that small sample volumes can be handled, solvent consumption was low, and the procedure was very fast (2 min per 96-well plate) (Table 4). Application of monolithic methacrylate polymer packed 96-tips in bioanalysis.

## 6. Conclusions

The advantages of various kinds of SPME, SBSE, and MEPS as powerful sample preparation methods in bioanalysis have been demonstrated and illustrated in many cases. It is clear that the number of the papers published in this research area has increased during the last decade. In addition, the results showed the ability of these techniques for determination of drugs in biological samples. Future work should be focused on finding of more selective, high throughput adsorption capacity and stable sorbents with capability for extraction of large molecules.

## Figures and Tables

**Table 1:** HSPME for analysis of different drug components.

Drug	Disease	Fiber type	Sample matrix	Analytical method	Reference
Dichlorobenzene	Damage to the liver and the kidneys	Polydimethylsiloxane	Human blood	GC/MS	[160]
Illegal drugs (acetylated amphetamine, secobarbital, phenobarbital, methadone, propoxyphene, imipramine, acetylated codeine, flunitrazepam, diacetylated morphine)	Polyacrylate	(In situ) derivatisation (acetylation or silylation)	Urine/serum	GC/MS	[88]
Thymol	Food/pharmaceuticals	Polydimethylsiloxane- divinylbenzene crimped fiber	Human plasma	GC/MS	[161]
Amphetamine-related drugs	Abused drugs	Polydimethylsiloxane	Human urine	GC/MS	[162]
Rivastigmine	Symptomatic treatment of mild to moderate dementia	Polydimethylsiloxane/divinylbenzene (pdms/dvb) or polydimethylsiloxane (pdms)	Canine plasma	GC/MS	[163]
Menthol	Flavouring agent	65 µm polydimethylsiloxane/divinylbenzene (PDMS/DVB)-coated fibers	Plasma/urine of rats	GC/MS	[90]
Ephedrine, methamphetamine	Anaesthesia psychoactive drug	Sol-gel	Human urine	GC	[34]
Asarones	Antiepileptic drugs	Polydimethylsiloxane (PDMS), 65 *μ*mm polydimethylsiloxane/divinylbenzene (PDMS/DVB), 65 *μ*mm carbowax/divinylbenzene (CW/DVB), 75 *μ*mm carboxen poly(dimethylsiloxane) (CAR/PDMS), 85 *μ*mm polyacrylate (PA),	Plasma	GC/MS	[164]
Paeonol	Eczema	100 µm polydimethylsiloxane (PDMS), 65 *μ*m polydimethylsiloxane/divinylbenzene (PDMS-DVB), 65 *μ*m carbowax/divinylbenzene (CW-DVB), 75 *μ*m carboxen poly(dimethylsiloxane) (CAR-PDMS), and 85 *μ*m polyacrylate (PA) were purchased from Supelco (Bellefonte, PA, USA).	Rabbit plasma /essential oil	GC/MS	[165]
Fentanyl	Surgical analgesia and sedation	Sol-gel technology	Human plasma	GC/MS	[166]
Diisopropylfluorophosphate	Miotic agent in treatment of chronic glaucoma	65 mm polydimethylsiloxane/divinylbenzene (pdms/dvb)	Rat plasma/brain tissue	GC/MS	[167]
Some phenothiazine derivatives	Antipsychotics (major tranquilizers), antiparkinsonism drugs, antihistaminics	100 *μ*m PDMS, 85 *μ*m polyacrylate, 65 *μ*m PDMS/divinylbenzene (DVB), 65 *μ*m Carbowax (CW)/DVB, 85 *μ*m stableflex carboxen (CAR)/PDMS, and 50/30 *μ*m stableflex DVB/CAR/PDMS	Human blood	GC/NPD	[168]
Methadone	Analgesic	Nanostructured a-carboxy Polypyrrole (ppy-*α*-COOH)	Plasma/urine	GC/FID	[169]
Ethyl glucuronide	Promising biomarker of heavy prenatal alcohol exposure	100 *μ*m polydimethylsiloxane red and black fibers	Human placenta	GC/MS	[170]
Ranitidine	Prescribed	Carboxen/polydimethylsiloxane	Solid state	GC/MS	[171]
Volatile organic metabolites	volatile organic metabolites	Pdms, pdms/dvb, cw/dvb, Pa, car/pdms, and dvb/car/pdms	Mentha ×piperita L	GC/qMS	[172]
*E. coli* BL21	Antimicrobial agents	For example, 100 *μ*m PDMS, 65 *μ*m DVB/PDMS, 75 *μ*m CAR/PDMS, and 50/30 *μ*m CAR/PDMS/DVB	Cell metabolism	GC/MS	[173]

**Table 2:** Function and chemical structures of the coating solution ingredients for sol-gel-derived DM-*β*-CD/OH-TSO coating [34].

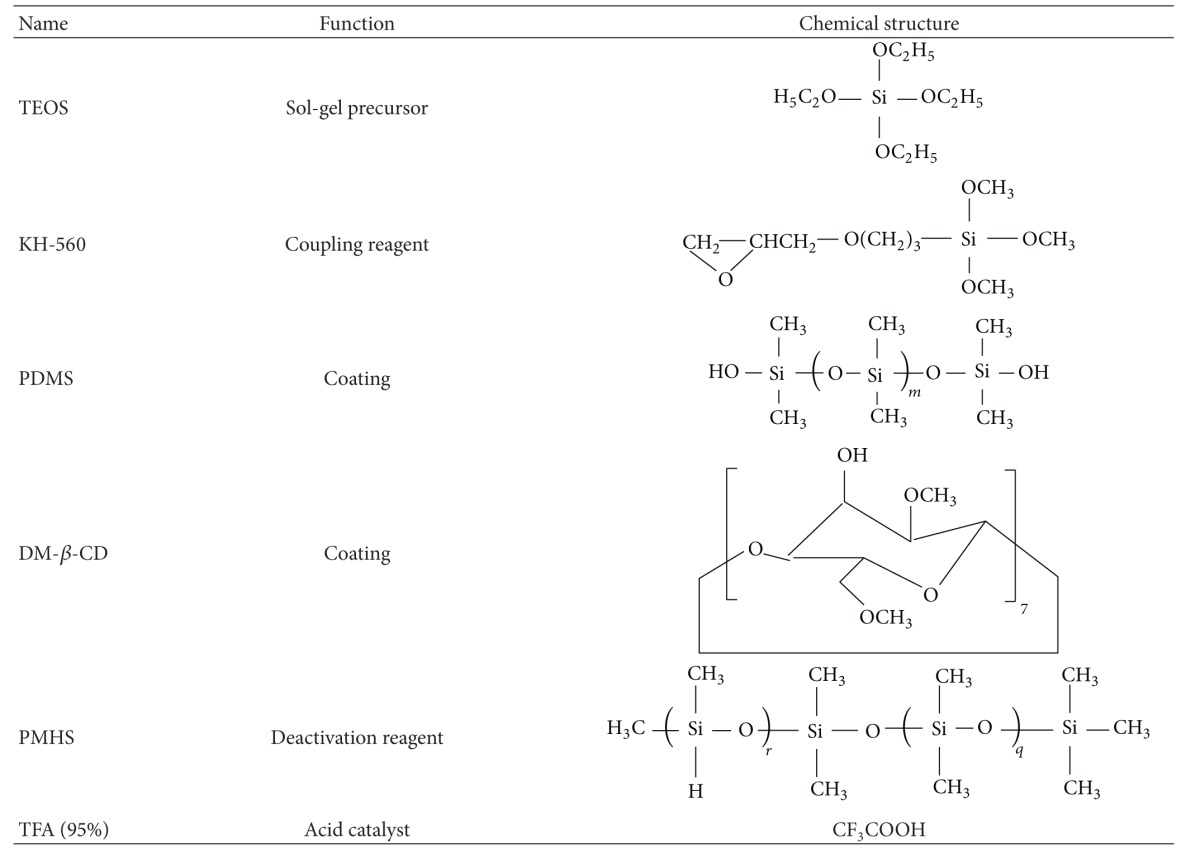

**Table 3:** Extraction of different analytes by MEPS technique.

Components	Sample matrix	MEPS sorbent	References
Methadone	Human urine	Silica-C8	[19]
Dopamine/serotonin	Human urine	Silica-C8	[20]
Cocaine/metabolites	Human urine	Silica-C8	[21]
Clozapine/metabolites	Dried blood spots	Silica-C8	[121]
Methadone/buprenorphine/norbuprenorphine/naloxone /levosulpiride	Plasma	Silica-C8	[122]
Pravastatin/pravastatin lactone	Rat plasma/urine	Silica-C8	[123]
Immunosuppressive drugs (Cyclosporine, everolimus, Sirolimus, tacrolimus)	Whole blood	Silica-C8	[124]
Oxcarbazepine/metabolites	Plasma/saliva	Silica-C18	[125]
Antiepileptic drugs	Human plasma/urine	Silica-C18	[126]
Amiodarone/desethylamiodarone	Human plasma	Silica-C18	[127]
Methadone	Dried blood spots	Silica-C18	[128]
Biogenic amines	Human urine	Silica-C18	[129]
Metabolites of monoterpenes	Human urine	C18/silica phases	[130]
Acebutolol, metoprolol	Human plasma/urine	Polystyrene polymer	[131]
Busulphan	Human plasma	Polystyrene polymer	[132]
Linezolid and amoxicillin	Human plasma	(C2, C8, C18, M1 (80% C8 and 20% SCX), and Sil (pure silicate))	[133]
Cotinine	Human urine	(C2, C8, C18, silica, and C8/SCX)	[134]
Psychotropic drugs	Human serum	C18, C8, and C8-SCX	[135]
Antipsychotic drugs	Human plasma	80% C8 and 20% SCX	[136]
Local anaesthetics	Human plasma	Benzenesulphonic acid cation exchange silica	[137]
Ropivacaine /metabolites	Human urine	Polystyrene polymer, ISOLUTE ENV+	[138]
Lidocaine, glycylxylidide (GX), monothylglycylxylidide (MEGX), and 3-OH lidocaine	Human plasma/urine	Silica based (C8), polymer based (ENV+), and a methacrylate based organic monolith	[139]
Antidepressants	Human plasma	C8/strong cationic exchange	[140]
Ropivacaine	Human plasma	Methylcyanopropyl/silarylene (50/50)	[141]
Ropivacaine, lidocaine, bupivacaine, mepivacaine	Human plasma	Molecularly imprinted polymers (mips)	[142, 147]
Cyclophosphamide	Human plasma	C2-sorbent	[143]
Codeine metabolites	Human urine	Barrel insert needle (BIN), poly(styrene co-divinylbenzene) (PS DVB)	[144]
Propranolol, metoprolol, verapamil	Human urine	C2, C8, C18, M1 (cation exchanger), and Sil (pure silica)	[145]
Bam peptide	Human plasma	C8	[148]

**Table 4:** Applications of monolithic methacrylate polymer packed 96-tip.

Compound class/compound	Sample matrix	Sample volume (*μ*L)	Analytical method	Calibration range	References
Local anaesthetics					
Lidocaine	Human plasma	100	LC-MS/MS	14–5000 nM	[154]
Ropivacaine	Human plasma	100	LC-MS/MS	2–2000 nM	[155]
Bupivacaine	Human plasma	100	LC-MS/MS	2–2000 nM	[156]
Anticancer drugs					
Cyclophosphamide	Mice blood	20	LC-MS/MS	10–2000 nM	[159]
Busulphan	Human blood	100	LC-MS	5–2000 nM	[159]
Roscovitine	Human plasma	100	LC-MS/MS	14–5000 nM	[154]
*β*-Blockers					
Pindolol	Human plasma	100	LC-MS/MS	0.5–5000 nM	[157, 158]
Metoprolol	Human plasma	100	LC-MS/MS	0.5–5000 nM	[157, 158]

## References

[1] Arthur CL, Pawliszyn J (1990). Solid phase microextraction with thermal desorption using fused silica optical fiber. *Analytical Chemistry*.

[2] Martinez Vidal JL, Plaza-Bolanos P, Romero-Gonzalez R, Garrido Frenich A (2009). Determination of pesticide transformation products: a review of extraction and detection methods. *Journal of Chromatography A*.

[3] Jelen HH, Majcher M, Dziadas M (2012). Microextraction techniques in the analysis of food flavor compounds: a review. *Analitica Chimica Acta*.

[4] Ouyang G, Vuckovic D, Pawliszyn J (2011). Nondestructive sampling of living systems using *in vivo* solid-phase microextraction. *Chemical Reviews*.

[5] Kataoka H (2010). Recent developments and applications of microextraction techniques in drug analysis. *Analytical and Bioanalytical Chemistry*.

[6] Pragst F (2007). Application of solid-phase microextraction in analytical toxicology. *Analytical and Bioanalytical Chemistry*.

[7] Eisert R, Pawliszyn J (1997). Design of automated solid-phase microextraction for trace analysis of organic compounds in aqueous samples. *Journal of Chromatography A*.

[8] Abdel-Rehim M, Andersson M, Portelius E, Norsten-Höög C, Blomberg L (2001). Determination of ropivacaine and its metabolites in human plasma using solid phase microextraction and GC-NPD / GC-MS. *Journal of Microcolumn Separations*.

[9] Abdel-Rehim M, Hassan Z, Blomberg L, Hassan M (2003). On-line derivatization utilizing solid-phase microextraction (SPME) for determination of busulphan in plasma using gas chromatography-mass spectrometry (GC-MS). *Therapeutic Drug Monitoring*.

[10] Zhang X, Es-haghi A, Musteata FM, Ouyang G, Pawliszyn J (2007). Quantitative *in vivo* microsampling for pharmacokinetic studies based on an integrated solid-phase microextraction system. *Analytical Chemistry*.

[11] Vuckovic D, Cudjoe E, Musteata FM, Pawliszyn J (2010). Automated solid-phase microextraction and thin-film microextraction for high-throughput analysis of biological fluids and ligand-receptor binding studies. *Nature Protocols*.

[12] Risticevic S, Chen Y, Kudlejova L (2010). Protocol for the development of automated high-throughput SPME-GC methods for the analysis of volatile and semivolatile constituents in wine samples. *Nature Protocols*.

[13] Lipinski J (2001). Automated solid phase dynamic extraction—extraction of organics using a wall coated syringe needle. *Fresenius’ Journal of Analytical Chemistry*.

[14] Lachenmeier DW, Kroener L, Musshoff F, Madea B (2003). Application of tandem mass spectrometry combined with gas chromatography and headspace solid-phase dynamic extraction for the determination of drugs of abuse in hair samples. *Rapid Communications in Mass Spectrometry*.

[15] Bagheri H, Ayazi Z, Sistani H (2011). Chemically bonded carbon nanotubes on modified gold substrate as novel unbreakable solid phase microextraction fiber. *Microchimica Acta*.

[16] Lenz D, Kröner L, Rothschild MA (2009). Determination of gamma-hydroxybutyric acid in serum and urine by headspace solid-phase dynamic extraction combined with gas chromatography-positive chemical ionization mass spectrometry. *Journal of Chromatography A*.

[17] Baltussen E, David F, Janssen H, Sandra P, Cramers C (1999). Stir bar sorptive extraction (SBSE), a novel extraction technique for aqueous samples: theory and principles. *Journal of Microcolumn Separations*.

[18] Abdel-Rehim M (2004). New trend in sample preparation: on-line microextraction in packed syringe for liquid and gas chromatography applications I. Determination of local anaesthetics in human plasma samples using gas chromatography-mass spectrometry. *Journal of Chromatography B*.

[19] El-Beqqali A, Abdel-Rehim M (2007). Quantitative analysis of methadone in human urine samples by microextraction in packed syringe-gas chromatography-mass spectrometry (MEPS-GC-MS). *Journal of Separation Science*.

[20] El-Beqqali A, Kussak A, Abdel-Rehim M (2007). Determination of dopamine and serotonin in human urine samples utilizing microextraction online with liquid chromatography/electrospray tandem mass spectrometry. *Journal of Separation Science*.

[21] Jagerdeo E, Abdel-Rehim M (2009). Screening of cocaine and its metabolites in human urine samples by direct analysis in real-time source coupled to time-of-flight mass spectrometry after online preconcentration utilizing microextraction by packed sorbent. *Journal of the American Society for Mass Spectrometry*.

[22] Abdel-Rehim M (2010). Recent advances in microextraction by packed sorbent for bioanalysis. *Journal of Chromatography A*.

[23] Krogh M, Johansen K, Cnnesen FT, Rasmussen KE (1995). Solid-phase microextraction for the determination of the free concentration of valproic acid in human plasma by capillary gas chromatography. *Journal of Chromatography B*.

[24] Ulrich S, Martens J (1997). Solid-phase microextraction with capillary gas-liquid chromatography and nitrogen-phosphorus selective detection for the assay of antidepressant drugs in human plasma. *Journal of Chromatography B*.

[25] Ugland HG, Krogh M, Rasmussen KE (1997). Aqueous alkylchloroformate derivatisation and solid-phase microextraction: determination of amphetamines in urine by capillary gas chromatography. *Journal of Chromatography B*.

[26] Krogh M, Grefslie H, Rasmussen KE (1997). Solvent-modified solid-phase microextraction for the determination of diazepam in human plasma samples by capillary gas chromatography. *Journal of Chromatography B*.

[27] Abdel-Rehim M, Carlsson G, Bielenstein M, Arvidsson T, Blomberg LG (2000). Evaluation of solid-phase microextraction for the study of protein binding in human plasma samples. *Journal of Chromatographic Science*.

[28] Vita M, Abdel-Rehim M, Nilsson C (2005). Stability, pKa and plasma protein binding of roscovitine. *Journal of Chromatography B*.

[29] García DDlC, Reichenbächer M, Danzer K, Hurlbeck C, Bartzsch C, Feller KH (1998). Analysis of wine bouquet components using headspace solid-phase microextraction-capillary gas chromatography. *Journal of Sepration Science*.

[30] de Oliveira MH, Queiroz MEC, Carvalho D, Silva SM, Lancas FM (2005). Determination of diazepam in human plasma by solid-phase microextraction and capillary gas chromatography-mass spectrometry. *Chromatographia*.

[31] Orellana-Velado NG, Pereiro R, Sanz-Medel A (2001). Solid phase microextraction gas chromatography-glow discharge-optical emission detection for tin and lead speciation. *Journal of Analytical Atomic Spectrometry*.

[32] Reubsaet KJ, Norli HR, Hemmersbach P, Rasmussen KE (1998). Determination of benzodiazepines in human urine and plasma with solvent modified solid phase micro extraction and gas chromatography; rationalisation of method development using experimental design strategies. *Journal of Pharmaceutical and Biomedical Analysis*.

[33] Luo F, Wu Z, Tao P, Cong Y (2009). Preparation by low-temperature nonthermal plasma of graphite fiber and its characteristics for solid-phase microextraction. *Analytica Chimica Acta*.

[34] Zhou J, Zeng Z (2006). Novel fiber coated with *β*-cyclodextrin derivatives used for headspace solid-phase microextraction of ephedrine and methamphetamine in human urine. *Analytica Chimica Acta*.

[35] Queiroz MEC, Silva SM, Carvalho D (2002). Determination of lamotrigine simultaneously with carbamazepine, carbamazepine epoxide, phenytoin, phenobarbital, and primidone in human plasma by SPME-GC-TSD. *Journal of Chromatographic Science*.

[36] Deng C, Zhang W, Zhang J, Zhang X (2004). Rapid determination of acetone in human plasma by gas chromatography-mass spectrometry and solid-phase microextraction with on-fiber derivatization. *Journal of Chromatography B*.

[37] Hu L, Chen DY (2009). Application of headspace solid phase microextraction for study of noncovalent interaction of borneol with human serum albumin. *Acta Pharmacologica Sinica*.

[38] Aresta A, Calvano CD, Palmisano F, Zambonin CG (2008). Determination of clenbuterol in human urine and serum by solid-phase microextraction coupled to liquid chromatography. *Journal of Pharmaceutical and Biomedical Analysis*.

[39] Silva BJG, Queiroz RHC, Queiroz MEC (2007). Simultaneous determination of nontricyclic antidepressants in human plasma by solid-phase microextraction and liquid chromatography (SPME-LC). *Journal of Analytical Toxicology*.

[40] Queiroz MEC, Silva SM, Carvalho D, Lanças FM (2002). Solid-phase microextraction-liquid chromatography (SPME-LC) determination of lamotrigine simultaneously with carbamazepine and carbamazepine 10, 11-epoxide in human plasma. *Journal of Se-Paration Science*.

[41] Fernandes C, Neto AJDS, Rodrigues JC, Alves C, Lanças FM (2007). Solid-phase microextraction-liquid chromatography (SPME-LC) determination of fluoxetine and norfluoxetine in plasma using a heated liquid flow through interface. *Journal of Chromatography B*.

[42] Katayama M, Matsuda YI, Shimokawa K (2001). Determination of *β*-blockers by high performance liquid chromatography coupled with solid phase microextraction from urine and plasma samples. *Analytical Letters*.

[43] Alves C, Fernandes C, Neto AJDS, Rodrigues JC, Queiroz MEC, Lanças FM (2006). Optimization of the SPME parameters and its online coupling with HPLC for the analysis of tricyclic antidepressants in plasma samples. *Journal of Chromatographic Science*.

[44] Aresta A, Palmisano F, Zambonin CG (2005). Determination of naproxen in human urine by solid-phase microextraction coupled to liquid chromatography. *Journal of Pharmaceutical and Biomedical Analysis*.

[45] Caris JA, Chaves AR, Queiroz MEC (2012). Evaluation of solid-phase microextraction using a polythiophene film and liquid chromatography with spectrophotometric detection for the determination of antidepressants in plasma samples. *Journal of the Brazilian Chemical Society*.

[46] Chaves AR, Júnior GC, Queiroz MEC (2009). Solid-phase microextraction using poly(pyrrole) film and liquid chromatography with UV detection for analysis of antidepressants in plasma samples. *Journal of Chromatography B*.

[47] Bojko B, Vuckovic D, Cudjoe E (2011). Determination of tranexamic acid concentration by solid phase microextraction and liquid chromatography-tandem mass spectrometry: first step to *in vivo* analysis. *Journal of Chromatography B*.

[48] Cantú MD, Toso DR, Lacerda CA, Lanças FM, Carrilho E, Queiroz MEC (2006). Optimization of solid-phase microextraction procedures for the determination of tricyclic antidepressants and anticonvulsants in plasma samples by liquid chromatography. *Analytical and Bioanalytical Chemistry*.

[49] Queiroz MEC, Oliveira EB, Breton F, Pawliszyn J (2007). Immunoaffinity in-tube solid phase microextraction coupled with liquid chromatography-mass spectrometry for analysis of fluoxetine in serum samples. *Journal of Chromatography A*.

[50] Alves C, Santos-Neto AJ, Fernandes C, Rodrigues JC, Lanças FM (2007). Analysis of tricyclic antidepressant drugs in plasma by means of solid-phase microextraction-liquid chromatography-mass spectrometry. *Journal of Mass Spectrometry*.

[51] Davis WC, Pol SSV, Schantz MM, Long SE, Day RD, Christopher SJ (2004). An accurate and sensitive method for the determination of methylmercury in biological specimens using GC-ICP-MS with solid phase microextraction. *Journal of Analytical Atomic Spectrometry*.

[52] Bianchi F, Careri M, Maffini M, Mangia A, Mucchino C (2006). Optimization of the solid phase microextraction procedure for the ultra-trace determination of organotin compounds by gas chromatography- inductively coupled plasma-mass spectrometry. *Journal of Analytical Atomic Spectrometry*.

[53] Yang L, Mester Z, Sturgeon RE (2002). Improvement in measurement precision with SPME by use of isotope dilution mass spectrometry and its application to the determination of tributyltin in sediment using SPME GC-ICP-MS. *Journal of Analytical Atomic Spectrometry*.

[54] Shen JX, Tama CI, Hayes RN (2006). Evaluation of automated micro solid phase extraction tips (*μ*-SPE) for the validation of a LC-MS/MS bioanalytical method. *Journal of Chromatography B*.

[55] Mester Z, Sturgeon RE, Lam JW (2000). Sampling and determination of metal hydrides by solid phase microextraction thermal desorption inductively coupled plasma mass spectrometry. *Journal of Analytical Atomic Spectrometry*.

[56] Vonderheide AP, Montes-Bayon M, Caruso JA (2002). Solid-phase microextraction as a sample preparation strategy for the analysis of seleno amino acids by gas chromatography-inductively coupled plasma mass spectrometry. *Analyst*.

[57] Tsoi Y-K, Tam S, Leung KS-Y (2010). Rapid speciation of methylated and ethylated mercury in urine using headspace solid phase microextraction coupled to LC-ICP-MS. *Journal of Analytical Atomic Spectrometry*.

[58] Olszowy P, Szultka M, Ligor T, Nowaczyk J, Buszewski B (2010). Fibers with polypyrrole and polythiophene phases for isolation and determination of adrenolytic drugs from human plasma by SPME-HPLC. *Journal of Chromatography B*.

[59] Rajabi AA, Yamini Y, Faraji M, Seidi S (2013). Solid-phase microextraction based on cetyltrimethylammonium bromide-coated magnetic nanoparticles for determination of antidepressants from biological fluids. *Medicinal Chemistry Research*.

[60] Aresta A, Monaci L, Zambonin CG (2002). Determination of delorazepam in urine by solid-phase microextraction coupled to high performance liquid chromatography. *Journal of Pharmaceutical and Biomedical Analysis*.

[61] Deng C, Li N, Ji J, Yang B, Duan G, Zhang X (2006). Development of water-phase derivatization followed by solid-phase microextraction and gas chromatography/mass spectrometry for fast determination of valproic acid in human plasma. *Rapid Communications in Mass Spectrometry*.

[62] Unceta N, Gomez-Caballero A, Sanchez A (2008). Simultaneous determination of citalopram, fluoxetine and their main metabolites in human urine samples by solid-phase microextraction coupled with high-performance liquid chromatography. *Journal of Pharmaceutical and Biomedical Analysis*.

[63] Alizadeh R, Najafi NM, Poursanic EMA (2012). Arrays of SnO_2_ nanorods as novel solid phase microextraction for trace analysis of antidepressant drugs in body fluids. *Journal of Pharmaceutical and Biomedical Analysis*.

[64] Buszewski B, Szultka M, Olszowy P, Bocian S, Ligor T (2011). A novel approach to the rapid determination of amoxicillin in human plasma by solid phase microextraction and liquid chromatography. *Analyst*.

[65] Olszowy P, Szultka M, Buszewski B (2011). Poly(3-alkylthiophenes): new sorption materials for solid phase microextraction of drugs isolated from human plasma. *Analytical and Bioanalytical Chemistry*.

[66] Olszowy P, Szultka M, Nowaczyk J, Buszewski B (2011). A new way of solid-phase microextraction fibers preparation for selected antibiotic drug determination by HPLC-MS. *Journal of Chromatography B*.

[67] de Oliveira ARM, Bonato PS (2007). Stereoselective determination of hydroxychloroquine and its major metabolites in human urine by solid-phase microextraction and HPLC. *Journal of Separation Science*.

[68] de Oliveira MH, Queiroz MEC, Carvalho D, Silva SM, Lanças FM (2005). Determination of diazepam in human plasma by solid-phase microextraction and capillary gas chromatography-mass spectrometry. *Chromatographia*.

[69] Wu J, Lord H, Kataoka H, Pawliszyn J (2000). Polypyrrole-coated capillary in-tube solid phase microextraction coupled with liquid chromatography-electrospray ionization mass spectrometry for the determination of *β*-blockers in urine and serum samples. *Journal of Microcolumn Separations*.

[70] Mullett WM, Levsen K, Borlak J, Wu J, Pawliszyn J (2002). Automated in-tube solid-phase microextraction coupled with HPLC for the determination of N-nitrosamines in cell cultures. *Analytical Chemistry*.

[71] Walles M, Mullett WM, Levsen K, Borlak J, Wunsch G, Pawliszyn J (2002). Verapamil drug metabolism studies by automated in-tube solid phase microextraction. *Journal of Pharmaceutical and Biomedical Analysis*.

[72] Jinno K, Kawazoe M, Saito Y, Takeichi T, Hayashida M (2001). Sample preparation with fiber-in-tube solid-phase microextraction for capillary electrophoretic separation of tricyclic antidepressant drugs in human urine. *Electrophoresis*.

[73] Yan LJ, Zhang QH, Feng YQ (2006). Octyl-functionalized hybrid silica monolithic column for reversed-phase capillary elec-trochromatography. *Journal of Chromatography A*.

[74] Zheng MM, Wang ST, Hu WK, Feng YQ (2010). In-tube solid-phase microextraction based on hybrid silica monolith coupled to liquid chromatography-mass spectrometry for automated analysis of ten antidepressants in human urine and plasma. *Journal of Chromatography A*.

[75] Jarmalaviciene R, Szumski M, Kornysova O (2008). Coupling of solid-phase microextraction continuous bed (monolithic) capillaries with capillary zone electrophoresis for direct analysis of drugs in biological fluids. *Electrophoresis*.

[76] Chaves AR, Silva BJG, Lanças FM, Queiroz MEC (2011). Biocompatible in-tube solid phase microextraction coupled with liquid chromatography-fluorescence detection for determination of interferon *α* in plasma samples. *Journal of Chromatography A*.

[77] Zhang SW, Xing J, Cai LS, Wu CY (2009). Molecularly imprinted monolith in-tube solid-phase microextraction coupled with HPLC/UV detection for determination of 8-hydroxy-2′;-deoxyguanosine in urine. *Analytical and Bioanalytical Chemistry*.

[78] Deng DL, Zhang JY, Chen C, Hou XL, Su YY, Wu L (2012). Monolithic molecular imprinted polymer fiber for recognition and solid phase microextraction of ephedrine and pseudoephedrine in biological samples prior to capillary electrophoresis analysis. *Journal of Chromatography A*.

[79] Zhang W, Chen Z (2013). Mussel inspired polydopamine functionalized poly(ether ether ketone) tube for online solid-phase microextraction-high performance liquid chromato-graphy and its application in analysis of protoberberine alkaloids in rat plasma. *Journal of Chromatography A*.

[80] Nie J, Zhang M, Fan Y, Wen Y, Xiang B, Feng YQ (2005). Biocompatible in-tube solid-phase microextraction coupled to HPLC for the determination of angiotensin II receptor antagonists in human plasma and urine. *Journal of Chromatography B*.

[81] Wen Y, Fan Y, Zhang M, Feng YQ (2005). Determination of camptothecin and 10-hydroxycamptothecin in human plasma using polymer monolithic in-tube solid phase microextraction combined with high-performance liquid chromatography. *Analytical and Bioanalytical Chemistry*.

[82] Wei F, Fan Y, Zhang M, Feng YQ (2005). Poly(methacrylic acid-ethylene glycol dimethacrylate) monolith in-tube solid-phase microextraction applied to simultaneous analysis of some amphetamine derivatives in urine by capillary zone electrophoresis. *Electrophoresis*.

[83] Yu QW, Wang X, Ma Q, Yuan BF, He HB, Feng YQ (2012). Automated analysis of non-steroidal anti-inflammatory drugs in human plasma and water samples by in-tube solid-phase microextraction coupled to liquid chromatography-mass spectrometry based on a poly(4-vinylpyridine-co-ethylene dimethacrylate) monolith. *Analytical Methods*.

[84] Melo LP, Queiroz RHC, Queiroz MEC (2011). Automated determination of rifampicin in plasma samples by in-tube solid-phase microextraction coupled with liquid chromatography. *Journal of Chromatography B*.

[85] Chaves AR, Queiroz MEC (2013). Immunoaffinity in-tube solid phase microextraction coupled with liquid chromatography with fluorescence detection for determination of interferon *α* in plasma samples. *Journal of Chromatography B*.

[86] Caris JA, Silva BJGA, Moises ECD, Lanchote VLC, Queiroz MENC (2012). Automated analysis of lidocaine and its metabolite in plasma by in-tube solid-phase microextraction coupled with LC-UV for pharmacokinetic study. *Journal of Separation Science*.

[87] Zhang Z, Pawliszyn J (1993). Headspace solid-phase microextraction. *Analytical Chemistry*.

[88] Staerk U, Kulpmann WR (2000). High-temperature solid-phase microextraction procedure for the detection of drugs by gas chromatography-mass spectrometry. *Journal of Chromatography B*.

[89] Bigham S, Medlar F, Kabir A, Shende C, Alli A, Malik A (2002). Sol-gel capillary microextraction. *Analytical Chemistry*.

[90] Spichiger M, Muhlbauer RC, Brenneisen R (2004). Determination of menthol in plasma and urine of rats and humans by headspace solid phase microextraction and gas chromatography-mass spectrometry. *Journal of Chromatography B*.

[91] Turiel E, Tadeo JL, Martin-Esteban A (2007). Molecularly imprinted polymeric fibers for solid-phase microextraction. *Analytical Chemistry*.

[92] Mullett WM, Martin P, Pawliszyn J (2001). In-tube molecularly imprinted polymer solid-phase microextraction for the selective determination of propranolol. *Analytical Chemistry*.

[93] Koster EHM, Crescenzi C, Hoedt WD, Ensing K, de Jong GJ (2001). Fibers coated with molecularly imprinted polymers for solid-phase microextraction. *Analytical Chemistry*.

[94] Hua X, Hu Y, Li G (2007). Development of novel molecularly imprinted solid-phase microextraction fiber and its application for the determination of triazines in complicated samples coupled with high-performance liquid chromatography. *Journal of Chromatography A*.

[95] Djozan D, Baheri T (2007). Preparation and evaluation of solid-phase microextraction fibers based on monolithic molecularly imprinted polymers for selective extraction of diacetylmorphine and analogous compounds. *Journal of Chromatography A*.

[96] Hu X, Hu Y, Li G (2007). Preparation and characterization of prometryn molecularly imprinted solid-phase microextraction fibers. *Analytical Letters*.

[97] Tan F, Zhao H, Li X (2009). Preparation and evaluation of molecularly imprinted solid-phase microextraction fibers for selective extraction of bisphenol A in complex samples. *Journal of Chromatography A*.

[98] Xu S, Zhang X, Suna Y, Yu D (2013). Microwave-assisted preparation of monolithic molecularly imprinted polymeric fibers for solid phase microextraction. *Analyst*.

[99] Qiu L, Liu W, Huang M, Zhang L (2010). Preparation and application of solid-phase microextraction fiber based on molecularly imprinted polymer for determination of anabolic steroids in complicated samples. *Journal of Chromatography A*.

[100] Huang J, Hu Y, Hu Y, Li G (2011). Development of metal complex imprinted solid-phase microextraction fiber for 2,2′ dipyridine recognition in aqueous medium. *Talanta*.

[101] Szultka M, Szeliga J, Jackowski M, Buszewski B (2012). Development of novel molecularly imprinted solid-phase microextraction fibers and their application for the determination of antibiotic drugs in biological samples by SPME-LC/MS. *Analytical and Bioanalytical Chemistry*.

[102] Ma C, Chen H, Sun N, Ye Y, Chen H (2012). Preparation of molecularly imprinted polymer monolith with an analogue of thiamphenicol and application to selective solid-phase microextraction. *Food Analytical Methods*.

[103] Hu Y, Pan J, Zhang K, Lian H, Li G (2013). Novel applications of molecularly-imprinted polymers in sample preparation. *TrAC Trends in Analytical Chemistry*.

[104] Hyotylainen T, Riekkola M-L (2008). Sorbent- and liquid-phase microextraction techniques and membrane-assisted extraction in combination with gas chromatographic analysis: a review. *Analytica Chimica Acta*.

[105] Kawaguchi M, Ito R, Saito K, Nakazawa H (2006). Novel stir bar sorptive extraction methods for environmental and biomedical analysis. *Journal of Pharmaceutical and Biomedical Analysis*.

[106] Liu W, Wang H, Guan Y (2004). Preparation of stir bars for sorptive extraction using sol-gel technology. *Journal of Chromatography A*.

[107] Kabir A, Furton KG, Malik A (2013). Innovations in sol-gel microextraction phases for solvent-free sample preparation in analytical chemistry. *TrAC Trends in Analytical Chemistry*.

[108] Yu C, Hu B (2009). Sol-gel polydimethylsiloxane/poly(vinylalcohol)-coated stir bar sorptive extraction of organophosphorus pesticides in honey and their determination by large volume injection GC. *Journal of Separation Science*.

[109] Yu C, Li X, Hu B (2008). Preparation of sol-gel polyethylene glycolpolydimethylsiloxane-poly(vinyl alcohol)-coated sorptive bar for the determination of organic sulfur compounds in water. *Journal of Chromatography A*.

[110] Hu Y, Zheng Y, Zhu F, Li G (2007). Sol-gel coated polydimethylsiloxane/*β*-cyclodextrin as novel stationary phase for stir bar sorptive extraction and its application to analysis of estrogens and bisphenol A. *Journal of Chromatography A*.

[111] Yu C, Hu B (2007). Novel combined stir bar sorptive extraction coupled with ultrasonic assisted extraction for the determination of brominated flame retardants in environmental samples using high performance liquid chromatography. *Journal of Chromatography A*.

[112] Martin-Esteban A (2013). Molecularly-imprinted polymers as a versatile, highly selective tool in sample preparation. *TrAC Trends in Analytical Chemistry*.

[113] Xu Z, Hu Y, Hu Y, Li G (2010). Investigation of ractopamine molecularly imprinted stir bar sorptive extraction and its application for trace analysis of *β*
_2_-agonists in complex samples. *Journal of Chromatography A*.

[114] Hu Y, Li J, Hu Y, Li G (2010). Development of selective and chemically stable coating for stir bar sorptive extraction by molecularly imprinted technique. *Talanta*.

[115] Hu Y, Li J, Li G (2011). Synthesis and application of a novel molecularly imprinted polymer-coated stir bar for microextraction of triazole fungicides in soil. *Journal of Separation Science*.

[116] Xu Z, Song C, Hu Y, Li G (2011). Molecularly imprinted stir bar sorptive extraction coupled with high performance liquid chromatography for trace analysis of sulfa drugs in complex samples. *Talanta*.

[117] Turiel E, Martin-Esteban A (2012). Molecularly imprinted stir bars for selective extraction of thiabendazole in citrus samples. *Journal of Separation Science*.

[118] Huang X, Yuan D (2007). Preparation of stir bars for sorptive extraction based on monolithic material. *Journal of Chromatography A*.

[119] Huang X, Qiu N, Yuan D, Huang B (2009). A novel stir bar sorptive extraction coating based on monolithic material for apolar, polar organic compounds and heavy metal ions. *Talanta*.

[120] Huang X, Lin J, Yuan D, Hu R (2009). Determination of steroid sex hormones in wastewater by stir bar sorptive extraction based on poly(vinylpyridine-ethylene dimethacrylate) monolithic material and liquid chromatographic analysis. *Journal of Chromatography A*.

[121] Saracino MA, Lazzara G, Prugnoli B, Raggi MA (2011). Rapid assays of clozapine and its metabolites in dried blood spots by liquid chromatography and microextraction by packed sorbent procedure. *Journal of Chromatography A*.

[122] Somaini L, Saracino MA, Marcheselli C, Zanchini S, Gerra G, Raggi MA (2011). Combined liquid chromatography-coulometric detection and microextraction by packed sorbent for the plasma analysis of long acting opioids in heroin addicted patients. *Analytica Chimica Acta*.

[123] Vlckova H, Rabatinová M, Miksova A (2012). Determination of pravastatin and pravastatin lactone in rat plasma and urine using UHPLC-MS/MS and microextraction by packed sorbent. *Talanta*.

[124] Said R, Pohanka A, Abdel-Rehim M, Beck O (2012). Determination of four immunosuppressive drugs in whole blood using MEPS and LC-MS/MS allowing automated sample work-up and analysis. *Journal of Chromatography B*.

[125] Saracino MA, Tallarico K, Raggi MA (2010). Liquid chromatographic analysis of oxcarbazepine and its metabolites in plasma and saliva after a novel microextraction by packed sorbent procedure. *Analytica Chimica Acta*.

[126] Rani S, Malik AK (2012). A novel microextraction by packed sorbent-gas chromatography procedure for the simultaneous analysis of antiepileptic drugs in human plasma and urine. *Journal of Separation Science*.

[127] Rodrigues M, Alvesc G, Rocha M, Queiroz J, Falcao A (2013). First liquid chromatographic method for the simultaneous determination of amiodarone and desethylamiodarone in human plasma using microextraction by packed sorbent (MEPS) as sample preparation procedure. *Journal of Chromatography B*.

[128] Saracino MA, Marcheselli C, Somaini L (2012). A novel test using dried blood spots for the chromatographic assay of methadone. *Analytical and Bioanalytical Chemistry*.

[129] Oppolzer D, Moreno I, da Fonseca B (2013). Analytical approach to determine biogenic amines in urine using microextraction in packed syringe and liquid chromatography coupled to electrochemical detection. *Biomedical Chromatography*.

[130] Matysik S, Matysik FM (2009). Microextraction by packed sorbent coupled with gas chromatography—mass spectrometry: application to the determination of metabolites of monoterpenes in small volumes of human urine. *Microchimica Acta*.

[131] El-Beqqali A, Kussak A, Blomberg L, Abdel-Rehim M (2007). Microextraction in packed syringe/liquid chromatography/electrospray tandem mass spectrometry for quantification of acebutolol and metoprolol in human plasma and urine samples. *Journal of Liquid Chromatography and Related Technologies*.

[132] Abdel-Rehim M, Hassan Z, Skansem P, Hassan M (2007). Simultaneous determination of busulphan in plasma samples by liquid chromatography-electrospray ionization mass spectrometry utilizing microextraction in packed syringe (MEPS) as on-line sample preparation method. *Journal of Liquid Chromatography and Related Technologies*.

[133] Szultka M, Krzeminski R, Szeliga J, Jackowski M, Buszewski B (2013). A new approach for antibiotic drugs determination in human plasma by liquid chromatography mass—spectrometry. *Journal of Chromatography A*.

[134] Lafay F, Vulliet E, Flament-Waton MM (2010). Contribution of microextraction in packed sorbent for the analysis of cotinine in human urine by GC-MS. *Analytical and Bioanalytical Chemistry*.

[135] Wietecha-Posłuszny R, Garbacik A, Woźniakiewicz M, Moos A, Wieczorek M, Kościelniak P (2012). Application of microextraction by packed sorbent to isolation of psychotropic drugs from human serum. *Analytical and Bioanalytical Chemistry*.

[136] da Fonseca BM, Moreno IED, Barroso M, Costa S, Queiroz JA, Gallar-do E (2013). Determination of seven selected antipsychotic drugs in human plasma using microextraction in packed sorbent and gas chromatography-tandem mass spectrometry. *Analytical and Bioanalytical Chemistry*.

[137] Altun Z, Abdel-Rehim M, Blomberg LG (2004). New trends in sample preparation: on-line microextraction in packed syringe (MEPS) for LC and GC applications—part III: determination and validation of local anaesthetics in human plasma samples using a cation exchange sorbent, and MEPS-LC-MS-MS. *Journal of Chromatography B*.

[138] Abdel-Rehim M, Dahlgren M, Blomberg L (2006). Quantification of ropivacaine and its major metabolites in human urine samples utilizing microextraction in a packed syringe automated with liquid chromatography-tandem mass spectrometry (MEPS-LC-MS/MS). *Journal of Separation Science*.

[139] Altun Z, Blomberg L, Jagerdeo E, Abdel-Rehim M (2006). Drug screening using microextraction in a packed syringe (MEPS)/mass spectrometry utilizing monolithic-, polymer-, and silica-based sorbents. *Journal of Liquid Chromatography and Related Technologies*.

[140] Chaves AR, Leandro FZ, Carris JA, Queiroz MEC (2010). Microextraction in packed sorbent for analysis of antidepressants in human plasma by liquid chromatography and spectrophotometric detection. *Journal of Chromatography B*.

[141] Abdel-Rehim M, Dahlgren M, Blomberg L, Claude S, Tabacchi R (2006). Microextraction in packed syringe (MEPS) utilizing methylcyanopropyl- silarylene as coating polymer for extraction of drugs in biological samples. *Journal of Liquid Chromatography and Related Technologies*.

[142] Abdel-Rehim M, Andersson LI, Altun Z, Blomberg LG (2006). Increasing sample preparation throughput using monolithic methacrylate polymer as packing material for 96-tip robotic device. *Journal of Liquid Chromatography and Related Technologies*.

[143] Said R, Hassan Z, Hassan M, Abdel-Rehim M (2008). Rapid and sensitive method for determination of cyclophosphamide in patients plasma samples utilizing microextraction by packed sorbent online with liquid chromatography-tandem mass spectrometry (MEPS-LC-MS/MS). *Journal of Liquid Chromatography and Related Technologies*.

[144] Candish E, Gooley A, Wirth HJ, Dawes PA, Shellie RA, Hilder EF (2012). A simplified approach to direct SPE-MS. *Journal of Separation Science*.

[145] Nielsen K, Lauritsen FR, Nissilä T, Ketola RA (2012). Rapid screening of drug compounds in urine using a combination of microextraction by packed sorbent and rotating micropillar array electrospray ionization mass spectrometry. *Rapid Communications in Mass Spectrometry*.

[146] Abdel-Rehim M (2011). Microextraction by packed sorbent (MEPS): a tutorial. *Analytica Chimica Acta*.

[147] Daryanavard SM, Jeppsson-Dadoun A, Andersson LI, Hashemi M, Colmjsö A, Abdel-Rehim M (2013). Molecularly imprinted polymer in microextraction by packed sorbent for the simultaneous determination of local anesthetics: lidocaine, ropivacaine, mepivacaine and bupivacaine in plasma and urine samples. *Biomedical Chromatography*.

[148] Ashri NY, Daryanavard M, Abdel-Rehim M (2013). Microextraction by packed sorbent and liquid chromatography-tandem mass spectrometry as a tool for quantification of peptides in plasma samples: determination of sensory neuron-specific receptors agonist BAM8-22 and antagonist BAM22-8 in plasma samples. *Biomedical Chromatography*.

[149] Abdel-Rehim A, Abdel-Rehim M (2013). Screening and determination of drugs in human saliva utilizing microextraction by packed sorbent and liquid chromatography-tandem mass spectrometry. *Biomedical Chromatography*.

[150] Abdel-Rehim M, Andersson A, Breitholtz-Emanuelsson A (2008). MEPS as a rapid sample preparation method to handle unstable compounds in a complex matrix: determination of AZD3409 in plasma samples utilizing MEPS-LC-MS-MS. *Journal of Chromatographic Science*.

[151] Said R, Pohanka A, Andersson M, Beck O, Abdel-Rehim M (2011). Determination of remifentanil in human plasma by liquid chromatography-tandem mass spectrometry utilizing micro extraction in packed syringe (MEPS) as sample preparation. *Journal of Chromatography B*.

[152] Abdel-Rehim M, Askemark Y, Norsten-Höög C, Pettersson KJ, Halldin M (2006). Quantification of 4-OH-2,6-xylidine and its conjugates in human urine samples utilising microextraction in packed syringe on-line with liquid chromatography and electrospray tandem mass spectrometry (MEPS-LC-MS/MS). *Journal of Liquid Chromatography and Related Technologies*.

[153] Said R, Kamel M, El-Beqqali A, Abdel-Rehim M (2010). Microextraction by packed sorbent for LC-MS/MS determination of drugs in whole blood samples. *Bioanalysis*.

[154] Altun Z, Blomberg LG, Abdel-Rehim M (2006). Increasing sample preparation throughput using monolithic methacrylate polymer as packing material for 96-tip robotic device. *Journal of Liquid Chromatography and Related Technologies*.

[155] Altun Z, Hjelmström A, Blomberg LG, Abdel-Rehim M (2008). Evaluation of monolithic packed 96-tips for solid-phase extraction of local anesthetics from human plasma for quantitation by liquid chromatography tandem mass spectrometry. *Journal of Liquid Chromatography and Related Technologies*.

[156] Altun Z, Hjelmström A, Abdel-Rehim M, Blomberg LG (2007). Surface modified polypropylene pipette tips packed with a monolithic plug of adsorbent for high-throughput sample preparation. *Journal of Separation Science*.

[157] Abdel-Rehim M, Persson C, Altun Z, Blomberg L (2008). Evaluation of monolithic packed 96-tips and liquid chromatography-tandem mass spectrometry for extraction and quantification of pindolol and metoprolol in human plasma samples. *Journal of Chromatography A*.

[158] Altun Z, Skoglund C, Abdel-Rehim M (2010). Monolithic methacrylate packed 96-tips for high throughput bioanalysis. *Journal of Chromatography A*.

[159] Skoglund C, Bassyouni F, Abdel-Rehim M (2013). Monolithic packed 96-tips set for high-throughput sample preparation: determination of cyclophosphamide and busulfan in whole blood samples by monolithic packed 96-tips and LC-MS. *Biomedical Chromatogrphy*.

[160] Liu J, Hara K, Kashimura S, Hamanaka T, Tomojiri S, Tanaka K (1999). Gas chromatographic-mass spectrometric analysis of dichlorobenzene isomers in human blood with headspace solid-phase microextraction. *Journal of Chromatography B*.

[161] Kohlert C, Abel G, Schmid E, Veit M (2002). Determination of thymol in human plasma by automated headspace solid-phase microextraction-gas chromatographic analysis. *Journal of Chromatography B*.

[162] Namera A, Yashiki M, Kojima T (2002). Automated headspace solid-phase microextraction and in-matrix derivatization for the determination of amphetamine-related drugs in human urine by gas chromatography-mass spectrometry. *Journal of Chromatographic Science*.

[163] Sha Y, Deng C, Liu Z, Huang T, Yang B, Duan G (2004). Headspace solid-phase microextraction and capillary gas chromatographic-mass spectrometric determination of rivastigmine in canine plasma samples. *Journal of Chromatography B*.

[164] Deng C, Lin S, Huang T, Duan G, Zhang X (2006). Development of gas chromatography/mass spectrometry following headspace solid-phase microextraction for fast determination of asarones in plasma. *Rapid Communications in Mass Spectrometry*.

[165] Dong L, Deng C, Wang J, Shen X (2007). Fast determination of paeonol in plasma by headspace solid-phase microextraction followed by gas chromatography-mass spectrometry. *Analytica Chimica Acta*.

[166] Bagheri H, Es-haghi A, Khalilian F, Rouini MR (2007). Determination of fentanyl in human plasma by head-space solid-phase microextraction and gas chromatography-mass spectrometry. *Journal of Pharmaceutical and Biomedical Analysis*.

[167] Xu M, Terry AV, Bartlett J, Bartlett MG (2008). Determination of diisopropylfluorophosphate in rat plasma and brain tissue by headspace solid-phase microextraction gas chromatography/mass spectrometry. *Rapid Communications in Mass Spectrometry*.

[168] Shinmen N, Lee XP, Kumazawa T (2008). Simultaneous determination of some phenothiazine derivatives in human blood by headspace solid-phase microextraction and gas chromatography with nitrogen-phosphorus detection. *Journal of Association of Official Analytical Chemists International*.

[169] Ebrahimzadeh H, Mehdinia A, Kamarei F, Moradi E (2012). A sensitive method for the determination of methadone in biological samples using nano-structured *α*-carboxy polypyrrol as a sorbent of SPME. *Chromatographia*.

[170] Matlow JN, Aleksa K, Lubetsky A, Koren G (2012). The detection and quantification of ethyl glucuronide in placental tissue and placental perfusate by headspace solid-phase microextraction coupled with gas chromatography-mass spectrometry. *Canadian Journal of Clinical Pharmacology*.

[171] Jamrogiewicz M, Wielgomas B (2013). Detection of some volatile degradation products released during photoexposition of ranitidine in a solid state. *Journal of Pharmaceutical and Biomedical Analysis*.

[172] Silva CL, Ccmara JS (2013). Profiling of volatiles in the leaves of *Lamiaceae* species based on headspace solid phase microextraction and mass spectrometry. *Food Research International*.

[173] Zakir Hossain SMZ, Bojko B, Pawliszyn J (2013). Silica-based ionic liquid coating for 96-blade system for extraction of aminoacids from complex matrixes. *Analytica Chimica Acta*.

